# A binding-activated CRISPR/dCas13X imaging system enables precise RNA tracking in plants

**DOI:** 10.1371/journal.pbio.3004043

**Published:** 2026-10-06

**Authors:** Jiayu Zhang, Yiran Tao, Yun Zhao, Miaomiao Lei, Yucheng Shen, Qicong Li, Chenxu Liu, Jiuyuan Bai

**Affiliations:** Key Laboratory of Bio-Resource and Eco-Environment of Ministry of Education, Microbiology and Metabolic Engineering Key Laboratory of Sichuan Province, College of Life Sciences, Sichuan University, Chengdu, China; MRC Laboratory of Molecular Biology, UNITED KINGDOM OF GREAT BRITAIN AND NORTHERN IRELAND

## Abstract

Visualizing the spatiotemporal dynamics of native RNA molecules with single-transcript-level sensitivity in living plant cells remains a formidable challenge due to the high background noise caused by the unique cellular architecture of plants. Here, using transiently transformed *Nicotiana benthamiana* cells and stable *Arabidopsis thaliana* lines, we present a high-signal-to-noise RNA imaging platform that couples CRISPR–dCas13X with a binding-associated fluorescence stabilization strategy. We identified a novel plant-specific degron that selectively targets unbound probes for proteasomal degradation while preserving target-associated signal in a manner consistent with binding-dependent protection, enabling high-fidelity detection of endogenous mRNA puncta with minimal perturbation. Leveraging this platform, we characterized dynamics of *FT* mRNA trafficking in living plant cells, including plasmodesmata-targeting, cell-to-cell trafficking, and long-distance movement. Loss-of-function analysis further identified the established RNA-binding protein GRP7 as a factor that contributes to efficient *FT* mRNA transport. This work establishes a useful toolkit for investigating plant RNA biology and provides mechanistic insight into the systemic transport of florigenic signals.

## Abstract 2

Visualizing native RNA dynamics in living plant cells at single transcript resolution is limited by high background fluorescence. This study develops a high signal-to-noise CRISPR-dCas13X imaging platform that overcomes this barrier and provides insights into mRNA trafficking dynamics.

## Introduction

Spatiotemporal regulation of gene expression is fundamental to multicellular life. While transcriptomic technologies provide high-throughput snapshots of RNA abundance, they fail to capture the dynamic life history of individual RNA molecules—from synthesis and processing to transport and decay [1–4]. In animal systems, live-cell single-molecule imaging has revolutionized our understanding of transcriptional bursting, subcellular localization, and synaptic plasticity [5–8]. However, translating these advances to plant biology remains a formidable challenge, largely due to the lack of robust, low-perturbation imaging tools capable of operating within the unique constraints of the plant cellular environment.

Current methods for live-cell RNA imaging rely on genetically encoded aptamer systems, such as MS2 and PP7. These methods recruit fluorescent coat proteins to arrays of stem-loops inserted into the target RNA [9,10]. Although powerful, these approaches typically require engineering target RNAs to carry long repetitive exogenous sequences, which may perturb mRNA stability, folding, or transport and raise concerns that the observed dynamics may reflect artifacts of the tag rather than the native behavior of the transcript [1,2,11].

The advent of CRISPR–Cas13 systems offers a “tag-free” alternative. Catalytically inactive Cas13 variants (dCas13) can be programmed by a CRISPR RNA (crRNA) to bind endogenous RNAs without altering their sequence [12–15]. When coupled with signal-amplification scaffolds like the SunTag, dCas13 platforms have enabled the visualization of low-abundance transcripts in mammalian cells [5,15]. However, transplanting this technology to plants has encountered a specific physical barrier: the vacuole. Unlike mammalian cells, mature plant cells are dominated by a large central vacuole that compresses the cytoplasm into a thin peripheral layer. This confinement drastically concentrates the pool of unbound fluorescent probes, creating a high-background “haze” that obscures single-molecule signals [16,17].

To overcome these limitations, we reasoned that improving the signal-to-noise ratio (SNR) requires a system that is not just “bright”, but “smart”—one that differentiates between bound and unbound states. While conditional protein degradation strategies have been explored in other systems [18–20], a dedicated, autonomously regulated instability module for plant RNA imaging has been lacking.

Here, we present a high-SNR, low-perturbation platform for single-transcript-level imaging of endogenous plant mRNAs. We engineered a “binding-activated” switch by identifying a plant-specific degron (cPIL) via proteomic screening and fusing it to a circularly permuted scFv. This design promotes the rapid degradation of unbound fluorescent probes, thereby enabling high-fidelity detection of endogenous RNA with minimal perturbation. We validated the system’s sensitivity using single-molecule FISH (smFISH) and demonstrated its versatility by tracking transcripts associated with root development. We further applied the platform to examine the dynamics of *FLOWERING LOCUS T* (*FT*) mRNA trafficking. Our observations extend previous evidence for *FT* mRNA mobility by providing live-cell visualization of plasmodesmata-associated puncta, cell-to-cell transfer events, and graft-associated long-distance transport. RNA pull-down and loss-of-function analyses further implicated the established RNA-binding protein GRP7, also termed AtSRBP1, as a factor contributing to efficient *FT* mRNA transport.

## Results

### Rational design of a GFP–cpScFv reporter for low-background RNA imaging

The CRISPR/dCas13 family-based SunTag systems have proven effective for endogenous RNA imaging in mammalian cells [17,21,22]. However, transferring these technologies to plant cells presents a unique physical barrier: the large central vacuole compresses the cytoplasm into a thin peripheral layer, which amplifies background noise by concentrating the pool of unbound scFv–GFP. This limitation, together with chloroplast autofluorescence, precludes high-contrast imaging (Fig 1A and 1B).

**Fig 1:**
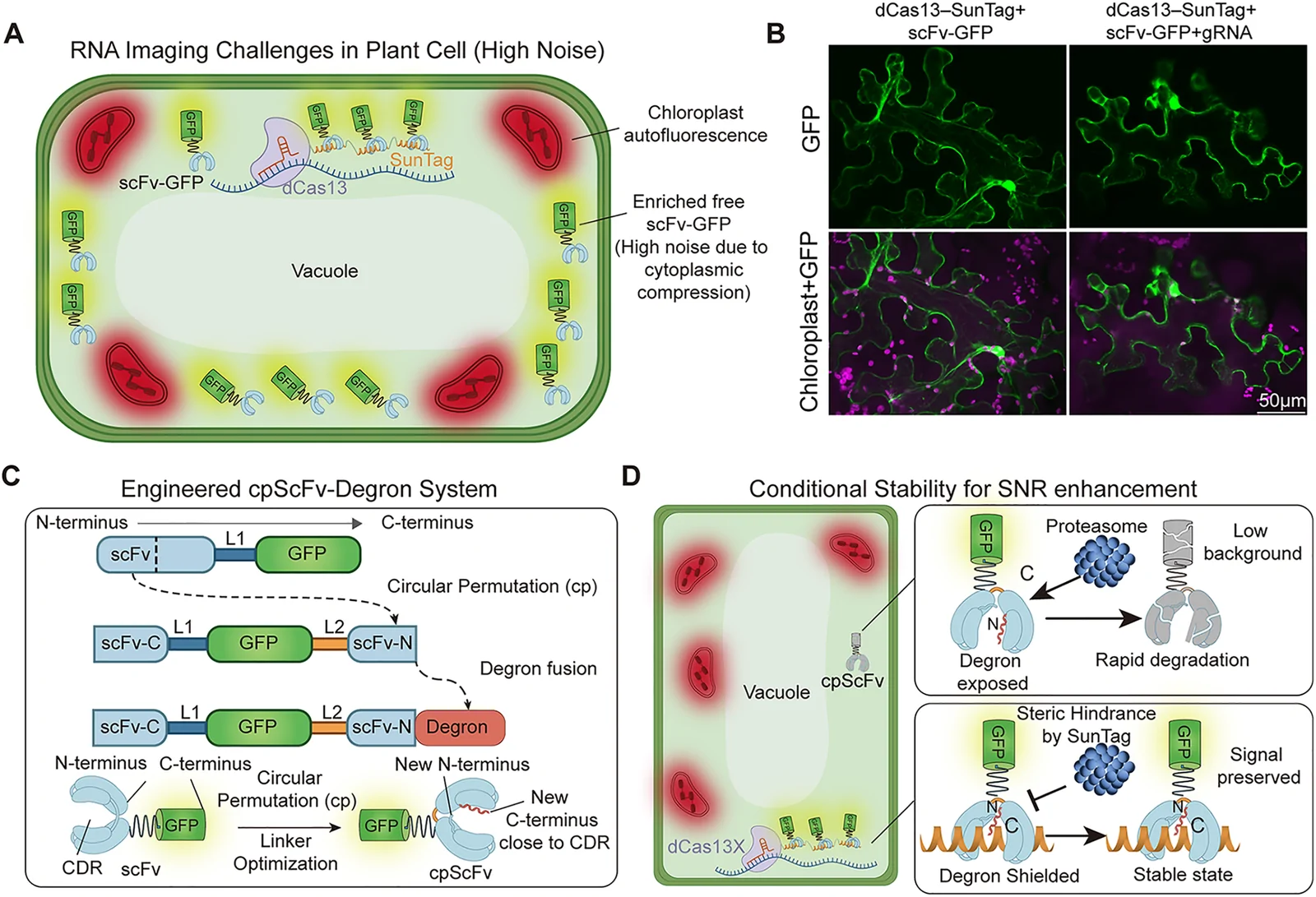
A conditional stability-based platform for high-signal-to-noise ratio (SNR) imaging of endogenous RNA in plants. **(A)** Schematic of the conventional dCas13–SunTag system. Background noise arises from the diffuse pool of unbound scFv–GFP in the cytoplasm. **(B)** Live-cell imaging of *NtACT7* mRNA in tobacco (*Nicotiana benthamiana*) leaf epidermal cells using the conventional dCas13–SunTag system revealed high background caused by the concentration of unbound scFv–GFP. Magenta indicates chloroplast autofluorescence. Scale bar, 50 μm. **(C)** Engineering the circularly permuted scFv (cpScFv) for conditional stability. Circular permutation relocates the N- and C-termini to the vicinity of the antigen-binding pocket, enabling the fusion of a degron (red tag) proximal to the binding interface. **(D)** Proposed model for ligand-dependent stabilization. In the unbound state, the exposed degron targets free GFP–cpScFv for proteasomal degradation, thereby reducing diffuse background. Upon binding to the SunTag array, the bound state is proposed to protect the degron from degradation machinery, stabilizing the fluorophore for high-contrast imaging.

To overcome this, we re-engineered the fluorescence module to function as a “binding-activated” switch. We reasoned that converting scFv–GFP from a constitutively stable reporter into a conditionally stable one would selectively deplete the unbound cytosolic pool. To this end, we designed a circularly permuted scFv (cpScFv) by relocating the N- and C-termini near the antigen-binding pocket, enabling the fusion of a degron proximal to the complementarity-determining region (CDR) (Fig 1C). Mechanistically, this cpScFv–degron module was designed to function as a binding-associated stabilization switch for proteasomal turnover. In the unbound state, the exposed degron promotes degradation of free GFP–cpScFv, thereby reducing diffuse background. Upon binding to the SunTag array, the bound state is proposed to protect the degron from degradation machinery, stabilizing the fluorophore at target-associated sites. Thus, RNA-dependent recruitment converts molecular recognition into selective signal retention, enabling high-contrast imaging of true RNA loci (Fig 1D).

### A proteome-wide screen identifies a plant-specific degron

Identifying a degron compatible with the plant cytosolic environment is critical. We first evaluated the efficiency of 10 previously reported degrons in *Nicotiana benthamiana* using a dual-fluorescence reporter system (Fig 2A). However, all tested degrons failed to sufficiently eliminate the GFP signal, with the lowest GFP/mCherry ratio remaining above 0.39 (Fig 2B and 2C), indicating insufficient instability for our purpose. Consequently, we performed a proteome-wide screen for endogenous instability domains. Using quantitative proteomics (LC–MS/MS) following MG132 treatment, we identified 86 proteins significantly upregulated upon proteasome inhibition and selected the top 30 candidates (Fig 2D). Screening the C-terminal peptides (CPs, 30 aa) of these candidates revealed that six peptides significantly induced GFP degradation (Figs 2E and S1). Among them, CP13—derived from a *Nicotiana tabacum* proteinase inhibitor I-B-like protein—exhibited the highest efficiency, suppressing the normalized GFP/mCherry ratio to <0.1 (Fig 2F). Truncation mapping further identified the C-terminal 21 amino acids as the core motif responsible for degradation (Fig 2G–2I). We designated this potent plant-specific degron as “cPIL” (C-terminal Proteinase Inhibitor-Like degron).

**Fig 2:**
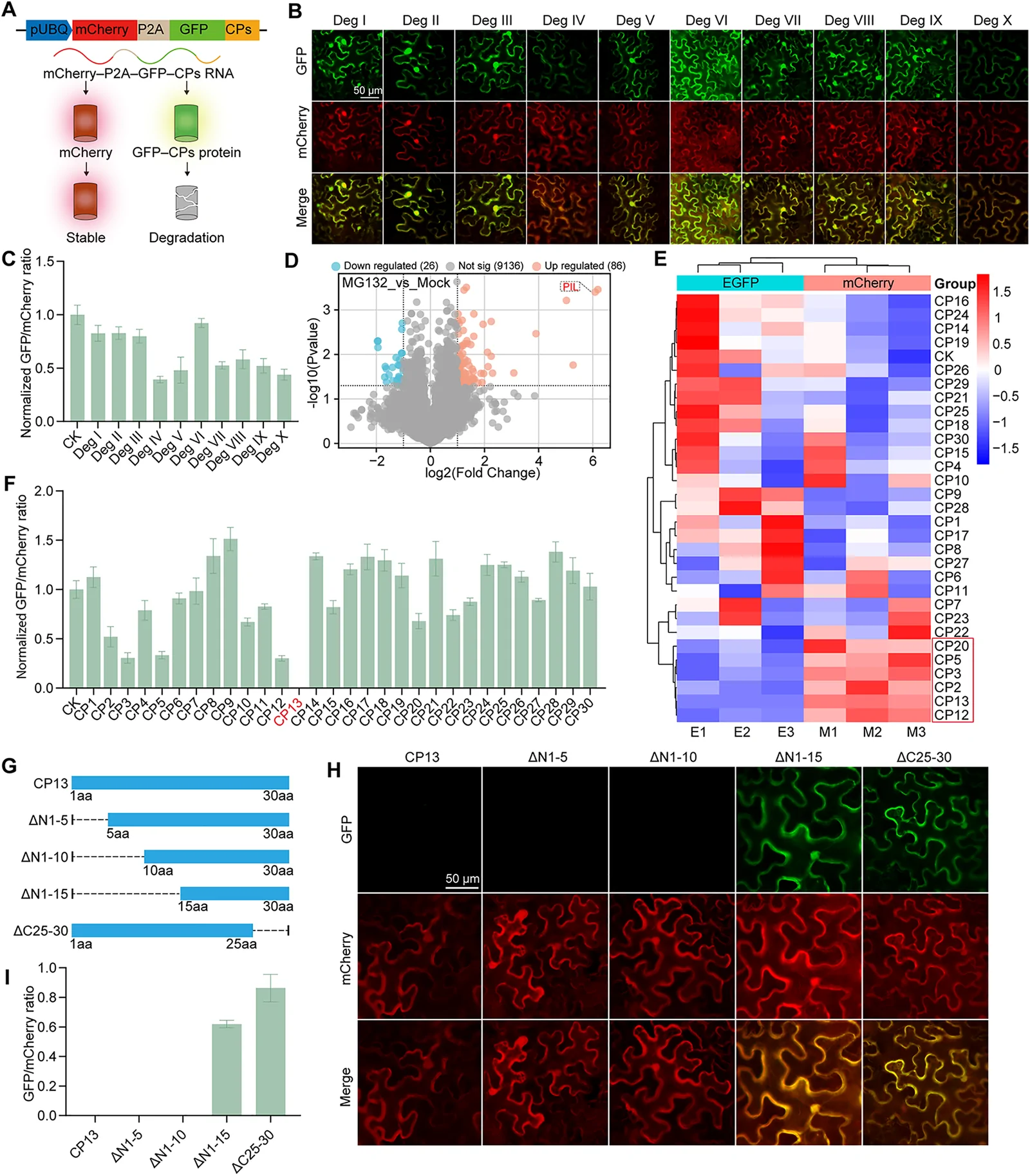
Proteome-wide screening identifies plant-specific C-terminal degrons. **(A)** Schematic of the dual-fluorescence reporter screen in *Nicotiana benthamiana*. A P2A self-cleaving peptide ensures stoichiometric expression of GFP (with candidate degrons) and mCherry (internal control). **(B)** Representative fluorescence images showing the stability of GFP fused with different degron tags. Scale bar, 50 μm. **(C)** Quantification of fluorescence ratios for indicated degrons. GFP/mCherry ratios were normalized to the control group (GFP–P2A–mCherry). **(D)** Volcano plot showing differentially expressed proteins identified by quantitative proteomics in samples treated with MG132 vs. Mock (PBS). **(E)** Differential fluorescence clustering analysis revealing the degradation efficiency of distinct C-terminal peptides. **(F)** Normalized GFP/mCherry fluorescence ratios for the indicated peptide candidates. **(G)** Schematic of CP13 truncation variants. **(H)** and **(I)** Representative images (H) and quantification (I) of GFP stability for CP13 truncations in *N. benthamiana* cells. Scale bar, 50 μm. All data are presented as mean ± s.d. (*n* = 3 biologically independent infiltrations). The data underlying this figure can be found in S1 Data.

### cPIL selectively depletes free reporters and enhances signal-to-noise in plant cells

To develop a SunTag-stabilized GFP–scFv reporter, we designed 10 circularly permuted GFP–scFv mutants with N- and C-termini near the antigen-binding pocket (Fig 3A). Imaging of tobacco epidermal cells co-expressing GFP–scFv mutants and SunTag revealed that only one mutant, with permuted termini adjacent to Asn194, produced fluorescent puncta when co-expressed with SunTag (Figs 3B and S2A). This mutant was designated “cpScFv”. The average number of puncta produced by GFP–cpScFv was comparable to that produced by wild-type GFP–scFv in SunTag-expressing cells (S2B Fig). These results indicate that cpScFv and wild-type scFv have similar SunTag-binding abilities.

**Fig 3:**
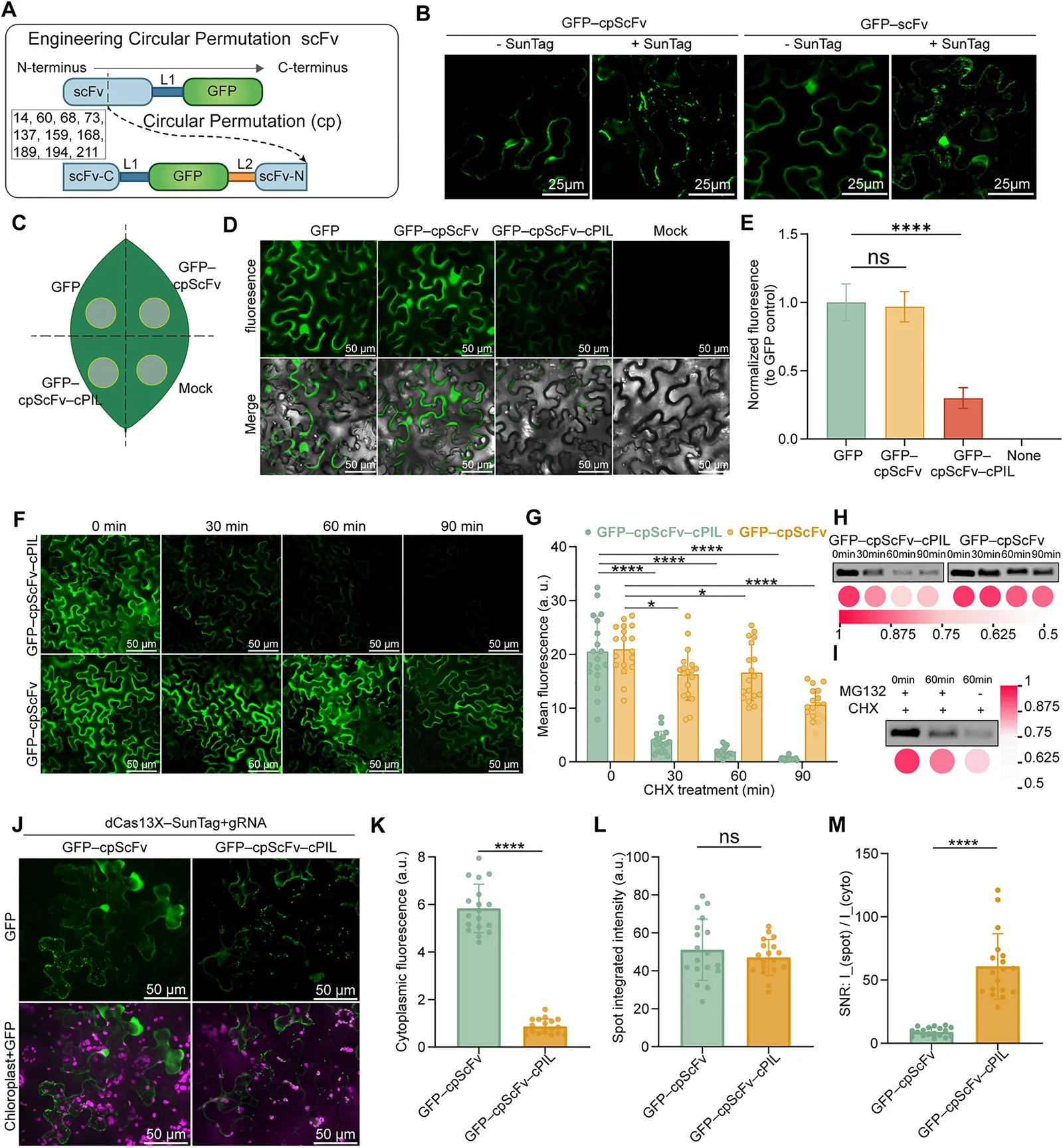
cPIL induces rapid degradation of GFP–cpScFv through the proteasome pathway. **(A)** Engineering the circularly permuted GFP–scFv reporter. Circular permutation relocates the N- and C-termini (indicated by number) to the vicinity of the antigen-binding pocket. **(B)** Imaging of tobacco epidermal cells showing the binding ability of GFP–cpScFv to SunTag. Scale bar, 25 μm. **(C)** Schematic of *Agrobacterium*-mediated infiltration in *N. benthamiana*. **(D)** Representative images of epidermal cells expressing the indicated vectors. Scale bar, 50 μm. **(E)** Quantification of fluorescence intensity corresponding to (D). Data are presented as mean ± s.d. (*n* = 3 biologically independent infiltrations). **(F)** and **(G)** Stability analysis under cycloheximide (CHX) treatment. Representative images (F) and time-course quantification (G) of GFP intensity in epidermal cells treated with 100 µM CHX for 0–90 min. Scale bar, 50 μm. Data are presented as mean ± s.d. (*n* = 18 cells from 3 biologically independent infiltrations). **(H)** and **(I)** Immunoblot analysis of GFP fusion protein abundance following treatment with CHX (H) or the proteasome inhibitor MG132 (I) for the indicated times. Relative protein levels are quantified below the blots using Fiji software. **(J)** Representative confocal images of *N. benthamiana* epidermal cells expressing dCas13X–24× SunTag with gRNA, combined with GFP–cpScFv or GFP–cpScFv–cPIL. Top: GFP channel; Bottom: Merged with chloroplast autofluorescence. Scale bar, 50 μm. **(K)** Quantification of cytosolic background intensity. Data are presented as mean ± s.d. (*n* = 18 cells from 3 biologically independent infiltrations). **(L)** Quantification of RNA puncta intensity. Data are presented as mean ± s.d. (*n* = 18 cells from 3 biologically independent infiltrations). **(M)** Signal-to-noise ratio (SNR), defined as mean puncta intensity divided by mean cytosolic neighboring intensity. Data are presented as mean ± s.d. (*n* = 18 cells from 3 biologically independent infiltrations). The data underlying this figure can be found in S1 Data. The uncropped blot and gel images are provided in S1 Raw Images.

We next examined whether the cPIL degron selectively destabilizes free GFP–cpScFv molecules in plant cells. Transient expression of either GFP–cpScFv or GFP–cpScFv–cPIL under the *UBQ10* promoter in the same *Nicotiana benthamiana* leaves revealed strong diffuse nuclear and cytoplasmic fluorescence for GFP–cpScFv and GFP control, whereas the cPIL fusion showed substantially reduced bulk signal, similar to the negative control (Fig 3C and 3D). Quantification data showed that cPIL caused a greater than 3.34-fold decrease in fluorescence intensity of GFP (Fig 3E). Time-lapse imaging showed an 80% decrease in fluorescence intensity of GFP–cpScFv–cPIL after 30 min of cycloheximide treatment, while the decrease in fluorescence in GFP–cpScFv-expressing cells was significantly attenuated over the same period (Fig 3F and 3G). Immunoblot analysis revealed that cPIL significantly reduced GFP-fusion protein accumulation compared with the cPIL-free control during cycloheximide chase (Fig 3H). These results indicate that cPIL promotes the rapid degradation of GFP–cpScFv. Furthermore, treatment with the proteasome inhibitor MG132 supported proteasome-dependent degradation of GFP–cpScFv–cPIL (Fig 3I).

To determine whether SunTag binding protects GFP–cpScFv–cPIL at RNA foci, we co-expressed full-length dCas13X–24× SunTag, a gRNA targeting the mCherry reporter mRNA, and either GFP–cpScFv or GFP–cpScFv–cPIL in a stable mCherry-expressing line. In both configurations, we detected discrete nuclear and cytoplasmic puncta corresponding to RNA–dCas13X–SunTag complexes (Fig 3J). Quantitative image analysis showed that the cPIL fusion reduced diffuse background fluorescence by approximately 6.71-fold relative to GFP–cpScFv, whereas the mean intensity of individual foci remained at a similar level (Fig 3K and 3L). Consequently, the SNR increased by approximately 6.67-fold in the GFP–cpScFv–cPIL system (Fig 3M). These observations support a model in which free GFP–cpScFv–cPIL molecules are rapidly removed by the proteasome, whereas SunTag binding stabilized GFP–cpScFv–cPIL.

### High-fidelity detection of endogenous *ACT2* mRNA puncta

We asked whether the GFP–cpScFv–cPIL reporter combined with the dCas13X–SunTag system was capable of imaging endogenous mRNAs in plant tissues. The cytoskeletal ACT2 transcript was selected as the target RNA. A single plasmid containing the *UBQ10::dCas13X–SunTag–IRES–GFP–cpScFv–cPIL–U6::gRNA* construct was introduced into wild-type Arabidopsis (Col-0), and three independent transgenic lines were used for imaging (Fig 4A). We observed discrete nuclear and cytoplasmic puncta in both leaf and root cells (Fig 4B).

**Fig 4:**
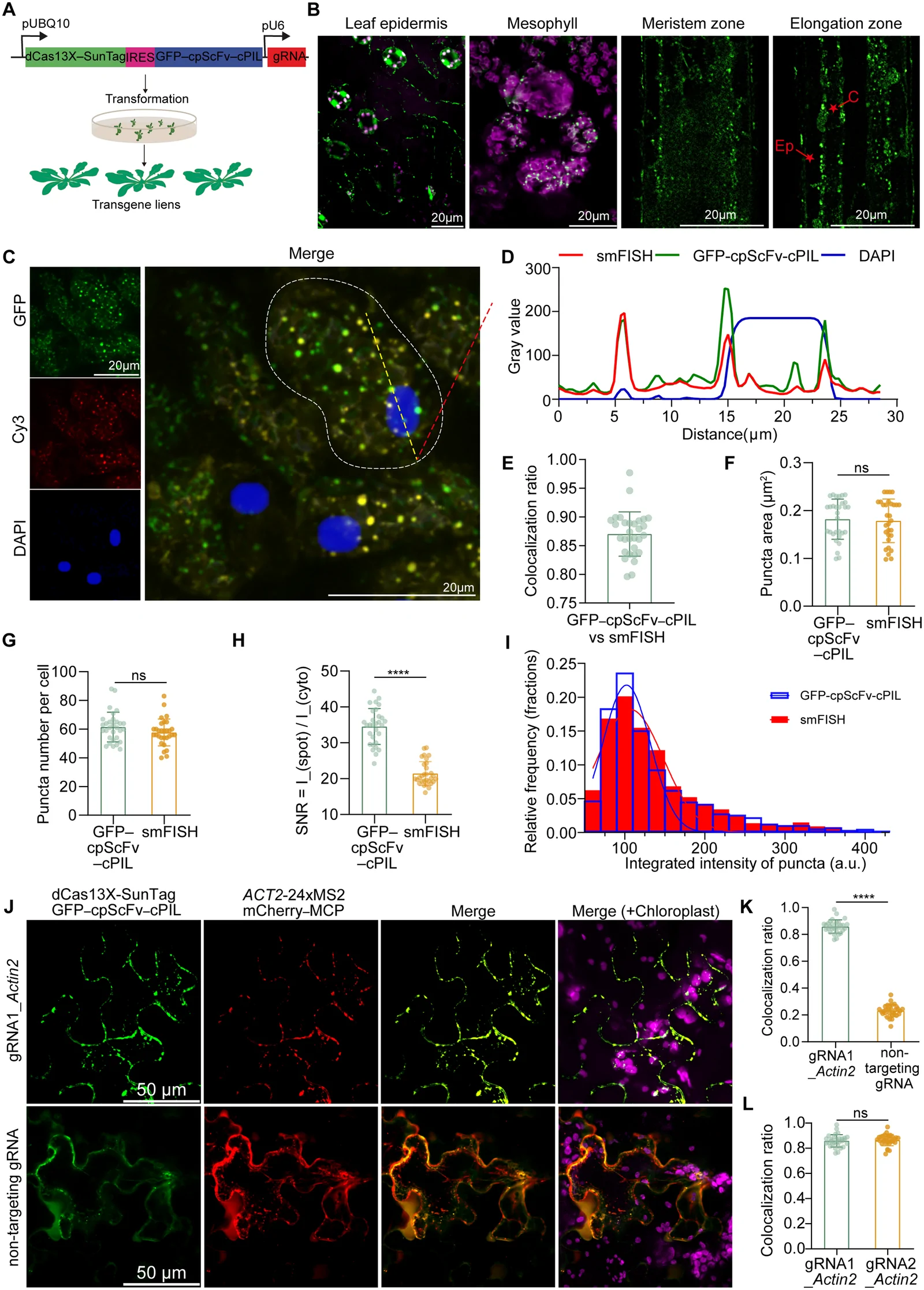
High-fidelity detection of endogenous *ACT2* RNA puncta. **(A)** Strategy for generating stable transgenic lines. **(B)** Live-cell imaging of *ACT2* mRNA in various tissues: leaf epidermis, mesophyll, root meristem, and elongation zone. Ep, epidermis; C, cortex. Scale bar, 20 μm. **(C)** Validation via single-molecule FISH (smFISH). *ACT2* mRNA was visualized by dCas13X–SunTag (green) and smFISH probes (red). Nuclei are stained with DAPI (blue). Right: Line-scan profiles of the dashed line. Scale bar, 20 μm. **(D)** Intensity profile of the region indicated by the yellow arrow in **(C)**, plotted with the X-axis in calibrated distance units (μm). **(E)** Colocalization ratio between dCas13X–SunTag and smFISH signals. **(F)** Puncta size detected by the dCas13X–SunTag system vs. smFISH. **(G)** Number of puncta per cell**. (H)** Comparison of signal-to-noise ratio (SNR) between the dCas13X–SunTag system and smFISH for high-fidelity detection of target RNA. **(I)** Quantification of integrated intensity distributions of individual *ACT2* puncta detected by the dCas13X–SunTag system (GFP) and by smFISH (Cy3). **(J)** Specificity assessment of the complete dCas13X–SunTag/GFP–cpScFv–cPIL imaging system using the *ACT2*–24× MS2 reporter assay. Tobacco epidermal cells co-expressed the *ACT2*–24× MS2 reporter RNA, MCP–mCherry, GFP–cpScFv–cPIL, dCas13X–SunTag, and either an *ACT2*-targeting gRNA or a non-targeting gRNA. GFP puncta indicate dCas13X–SunTag/GFP–cpScFv–cPIL signals, whereas mCherry puncta indicate MCP-bound *ACT2*–24× MS2 reporter RNA. The non-targeting gRNA condition retains the complete imaging and reporter system and serves as the OFF-state control for guide-dependent target recognition. Scale bar, 50 μm. **(K)** and **(L)** Quantification of colocalization between dCas13X–SunTag/GFP–cpScFv–cPIL puncta and MCP–mCherry foci using the non-targeting gRNA, *ACT2*-targeting gRNA1, or *ACT2*-targeting gRNA2. Colocalization was calculated as the fraction of GFP puncta overlapping with MCP–mCherry foci. All data are presented as mean ± s.d. (*n* = 30 cells from 3 biologically independent samples). The data underlying this figure can be found in S1 Data.

To confirm the single-transcript-level sensitivity, we benchmarked the GFP–cpScFv–cPIL system against smFISH in fixed leaves from the transgenic lines. We observed strong colocalization between GFP–cpScFv–cPIL puncta and smFISH puncta (Fig 4C and 4D), with an overall colocalization rate of 87.0 ± 3.8% (Fig 4E). GFP–cpScFv–cPIL showed a punctum-area distribution (0.1 μm^2^ to 0.23 μm^2^, consistent with the theoretical resolution limit of single-molecule RNA) and numbers of puncta per cell similar to those detected by smFISH (Fig 4F and 4G), while GFP–cpScFv–cPIL exhibited higher SNR than smFISH (Fig 4H), indicating improved imaging contrast for detection of endogenous RNA puncta in this assay. To further evaluate the nature of these puncta, we quantified the integrated intensity distributions of individual *ACT2* dCas13X–SunTag puncta and corresponding *ACT2* smFISH puncta. The *ACT2* dCas13X–SunTag puncta were dominated by a major low-intensity population with a progressively decreasing higher-intensity tail, and this overall distribution pattern was broadly similar to that observed for *ACT2* smFISH puncta (Fig 4I). Together, these data support the interpretation that most detected puncta represent single-transcript events under our assay conditions.

Furthermore, the specificity of GFP–cpScFv–cPIL for RNA imaging was validated using an MS2/MCP reporter system, in which a 24× MS2 tag was inserted into the 3′ UTR of *ACT2* RNA to recruit mCherry-MCP. In tobacco epidermal cells co-expressing *ACT2*–24× MS2 reporter RNA, MCP–mCherry, GFP–cpScFv–cPIL, dCas13X–SunTag, and gRNA1, 85.8 ± 4.9% of GFP–cpScFv–cPIL puncta colocalized with MCP–mCherry foci, while a non-targeting gRNA markedly reduced the colocalization between dCas13X–SunTag puncta and MCP–mCherry foci (Fig 4J and 4K), indicating that signal detection is guide dependent. In addition, two independent *ACT2*-targeting gRNAs produced similarly high colocalization with the MCP–mCherry reporter (Figs 4L and S3), supporting reproducible detection across independent guides. Together, these results strengthen the specificity of the system.

Next, we assessed whether the GFP–cpScFv–cPIL system perturbed the target RNA. Measurements of ACT2 transcript abundance and protein levels in transgenic *Arabidopsis thaliana* carrying either the reporter system or the empty-vector control revealed that our system had no significant effect on target-gene transcript or protein abundance (S4A and S4B Fig). The RNA decay curve after treatment with actinomycin D indicated that the reporter system had no significant effect on the turnover of the target mRNA (S4C Fig). Collectively, these data support GFP–cpScFv–cPIL as an effective platform for RNA imaging with single-transcript-level sensitivity in plant cells.

### Validation of generalizability across diverse endogenous targets

To evaluate the broad applicability of the dCas13X–SunTag/GFP–cpScFv–cPIL platform to diverse classes of endogenous plant mRNAs, we utilized the *Arabidopsis* root as an in vivo model to image endogenous mRNAs with distinct spatiotemporal profiles. We selected *CORTEX4*, *IRX9*, and *ATPP2-A1*—genes exhibiting cell-type–biased expression in the cortex, xylem, and phloem, respectively—as representative targets. For each gene, guide RNAs (gRNAs) targeting accessible regions within the coding sequence (CDS) were co-expressed with the optimized imaging module (Fig 5A–5C).

**Fig 5:**
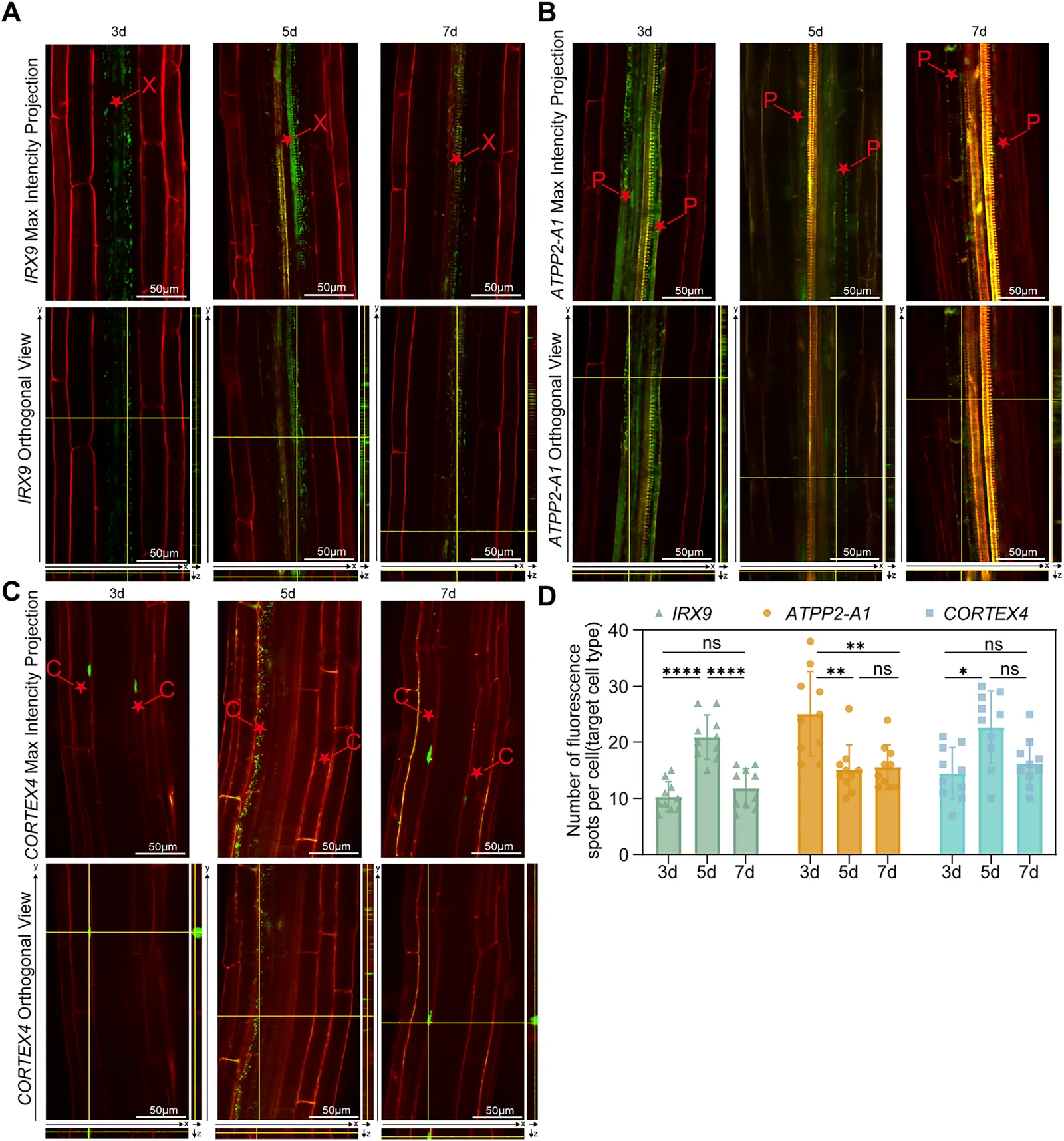
Cell-type-restricted visualization of endogenous mRNAs in *Arabidopsis* roots using the dCas13X–SunTag/GFP–cpScFv–cPIL platform. **(A–C)** Representative confocal images showing endogenous *IRX9* **(A)**, *ATPP2-A1* **(B)**, and *CORTEX4* (C) mRNA puncta in *Arabidopsis* roots at 3, 5, and 7 days after germination (DAG). *IRX9*, *ATPP2-A1*, and *CORTEX4* were selected as representative transcripts enriched in xylem, phloem-domain, and cortical cells, respectively. Green indicates dCas13X–SunTag/GFP–cpScFv–cPIL signals corresponding to RNA puncta, and red indicates propidium iodide (PI)-stained cell walls, which provide anatomical context for cell-type assignment. Upper panels show maximum-intensity projections; lower panels show orthogonal views used to verify the spatial localization of RNA puncta within the corresponding root cell files. X, xylem; P, phloem-domain cells; C, cortex. Red labels indicate representative target cell domains. Scale bars, 50 μm. **(D)** Quantification of fluorescent RNA puncta per cell at the indicated developmental stages. Quantification was restricted to the relevant target cell type for each marker: xylem cells for *IRX9*, phloem-domain cells for *ATPP2-A1*, and cortical cells for *CORTEX4*. Data are presented as mean ± s.d. (n = 10 cells per time point from 3 biologically independent roots). Statistical significance was determined by one-way ANOVA followed by Tukey’s multiple-comparisons test; ns, not significant; *P < 0.05, **P < 0.01, ****P < 0.0001. The data underlying this figure can be found in S1 Data.

Confocal microscopy revealed discrete, diffraction-limited fluorescent foci for all three targets, which were clearly resolvable at the single-cell level. The spatial distribution of these foci was highly specific and concordant with established transcriptomic atlases [23–25]. *CORTEX4* signals were enriched in cortical cell files, while *IRX9* and *ATPP2-A1* signals were strictly localized to the vascular domains (Fig 5A–5C). Quantitative analysis performed within the relevant target cell type for each marker confirmed that the platform captures expression dynamics across developmental stages (3, 5, and 7 days after germination [DAG]) (Fig 5D). Specifically, quantification was carried out in xylem cells for *IRX9*, phloem-domain cells for *ATPP2-A1*, and cortical cells for *CORTEX4*. Collectively, these results demonstrate that the platform enables high-contrast, cell-type–specific visualization of endogenous transcripts in intact plant tissues.

### Visualizing the long-distance trafficking of *FT* mRNA

Finally, we applied our platform to further examine the dynamics of *FLOWERING LOCUS T* (*FT*) mRNA trafficking in living plant cells. We generated two types of transgenic lines expressing dCas13X–SunTag/GFP–cpScFv–cPIL with and without gRNA in wild-type backgrounds. We observed obvious colocalization of *FT* mRNA puncta with the PDCB1-BFP-indicated plasmodesmata (Fig 6A). Furthermore, time-lapse imaging showed rapid translocation between cells after docking at plasmodesmata-associated regions (Figs 6B and S5; S1 and S2 Videos). Quantitative analysis of *FT* mRNA punctum dynamics during cell-to-cell trafficking revealed that *FT* puncta typically first accumulated at plasmodesmata-associated regions, where they exhibited a relatively long mean dwell time of approximately 20 s before transfer (Fig 6C; S1 and S2 Videos). This residence phase was followed by a rapid intercellular passage step, with an average transit time of approximately 6 s across the cell boundary (Fig 6D; S1 and S2 Videos). Quantification of trafficking frequency further revealed an average of approximately 12 successful transfer events per minute per cell interface (Fig 6E). Together, these kinetic measurements suggest that *FT* mRNA trafficking involves at least two distinguishable phases: an initial residence phase at plasmodesmata-associated regions followed by rapid intercellular passage.

**Fig 6:**
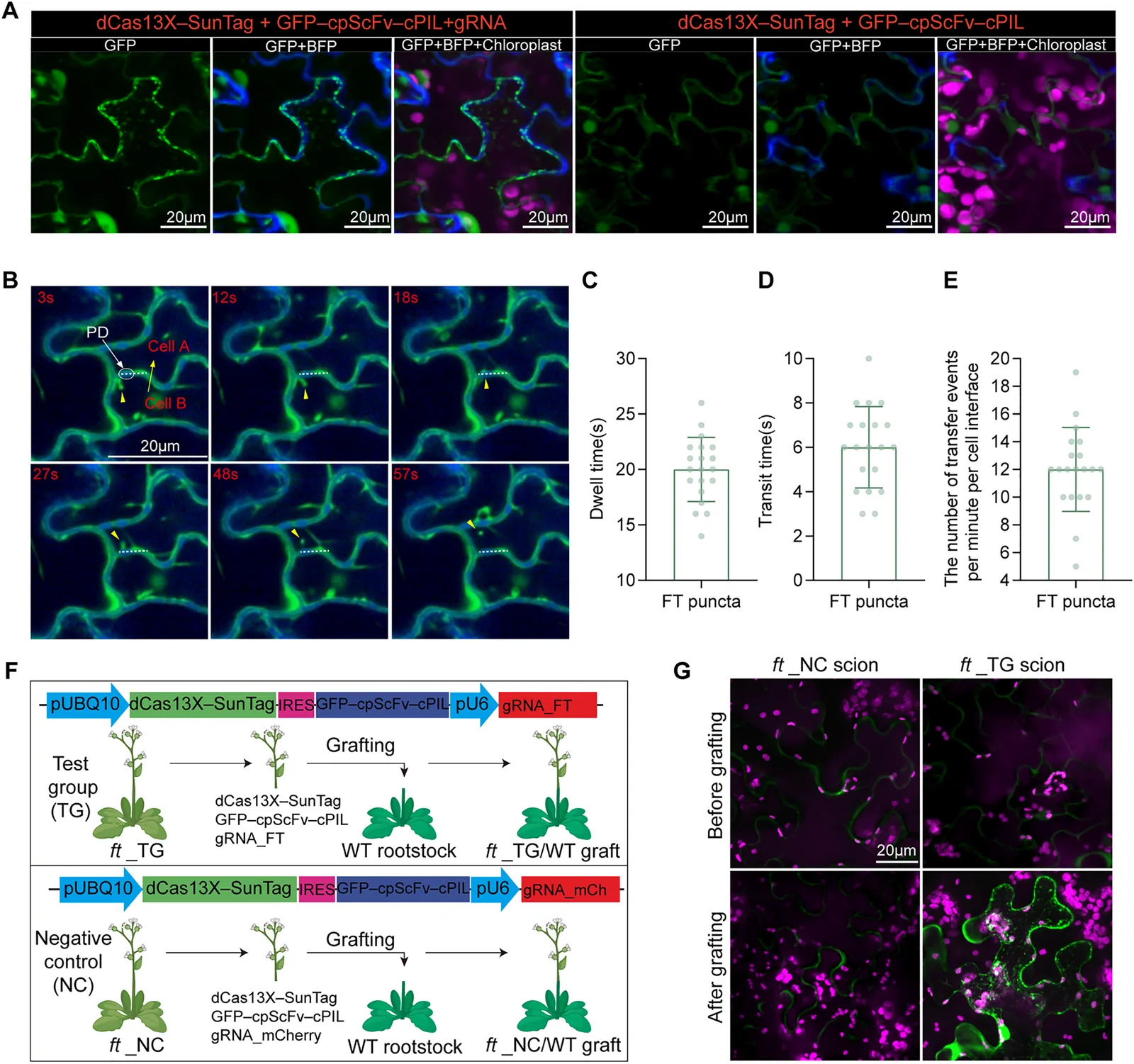
Visualization of long-distance *FT* mRNA trafficking across graft unions. **(A)** Representative image showing *FT* mRNA targeting plasmodesmata (PD). Scale bars, 20 μm. Green puncta represent *FT* mRNA visualized with the dCas13X–SunTag/GFP–cpScFv–cPIL system. Plasmodesmata (PD) are indicated by PDCB1–BFP (blue). **(B)** Time-lapse imaging capturing the cell-to-cell movement of *FT* mRNA in epidermal cells. The plasmodesmata are marked by PDCB1–BFP (blue). Scale bar, 20 μm. **(C)** Quantification of *FT* mRNA dwell time at plasmodesmata-associated regions prior to intercellular transfer. **(D)** Quantification of *FT* mRNA transit time across the cell boundary during successful transfer events. **(E)** Frequency of successful *FT* mRNA transfer events, expressed as the number of events per minute per cell interface. **(F)** Schematic of the grafting assay using different transgenic lines to visualize long-distance transport of *FT* mRNA. The *ft*_TG lines were *ft* mutant plants expressing dCas13X–SunTag/GFP–cpScFv–cPIL and an *FT*-targeting gRNA. The control lines were *ft* mutant plants expressing dCas13X–SunTag/GFP–cpScFv–cPIL and a non-*FT*-targeting gRNA. **(G)** Representative images of scions before and after grafting showing *FT* mRNA transport from the WT rootstock to the transgenic *ft* scion. Scale bars, 20 μm. Data in (C–E) are presented as mean ± s.d. For dwell-time and transit-time analyses in **(C)** and **(D)**, *n* = 20 transfer events from 6 cell interfaces in 3 biologically independent plants. For transfer-frequency analysis in **(E)**, *n* = 20 cell interfaces from 3 biologically independent plants. The data underlying this figure can be found in S1 Data.

To further test long-distance transport, we generated two types of transgenic lines expressing dCas13X–SunTag/GFP–cpScFv–cPIL and either an *FT*-targeting or a non-targeting gRNA in transcript-null *ft* mutant *Arabidopsis* plants (*ft*_TG and *ft*_NC lines), which were grafted onto WT rootstocks (Fig 6F). We observed no fluorescent puncta in either *ft*_TG or *ft*_NC scions before grafting, whereas fluorescent puncta emerged in *ft*_TG scions but remained absent from *ft*_NC scions two weeks after grafting (Fig 6G), supporting the long-distance movement of *FT* mRNA from the WT rootstock to the *ft* mutant scion. The presence of full-length *FT* mRNA in the scion was confirmed by RT-PCR and sequencing (S6A and S6B Fig). Together, these data provide visual evidence supporting rootstock-to-scion *FT* mRNA transport.

### GRP7 functionally contributes to efficient *FT* mRNA transport

To explore factors that may contribute to *FT* mRNA trafficking, we performed RNA pull-down assays using in vitro-transcribed, biotin-labeled full-length *FT* sense RNA, with antisense RNA as a control, followed by LC–MS/MS analysis of proteins enriched in the *FT* RNA pull-down (Fig 7A and 7B). From the differentially enriched candidates, we focused on three *FT* RNA-associated proteins: GLYCINE-RICH PROTEIN 7 (GRP7, AT2G21660), FRIGIDA-ESSENTIAL 1 (FES1, AT2G33835), and SWELLMAP-LIKE (SMPL, AT3G45950) (Fig 7C). Immunoblot analysis after RNA pull-down further confirmed enrichment of these candidate proteins in the *FT* RNA pull-down relative to the antisense RNA control (Fig 7D).

**Fig 7:**
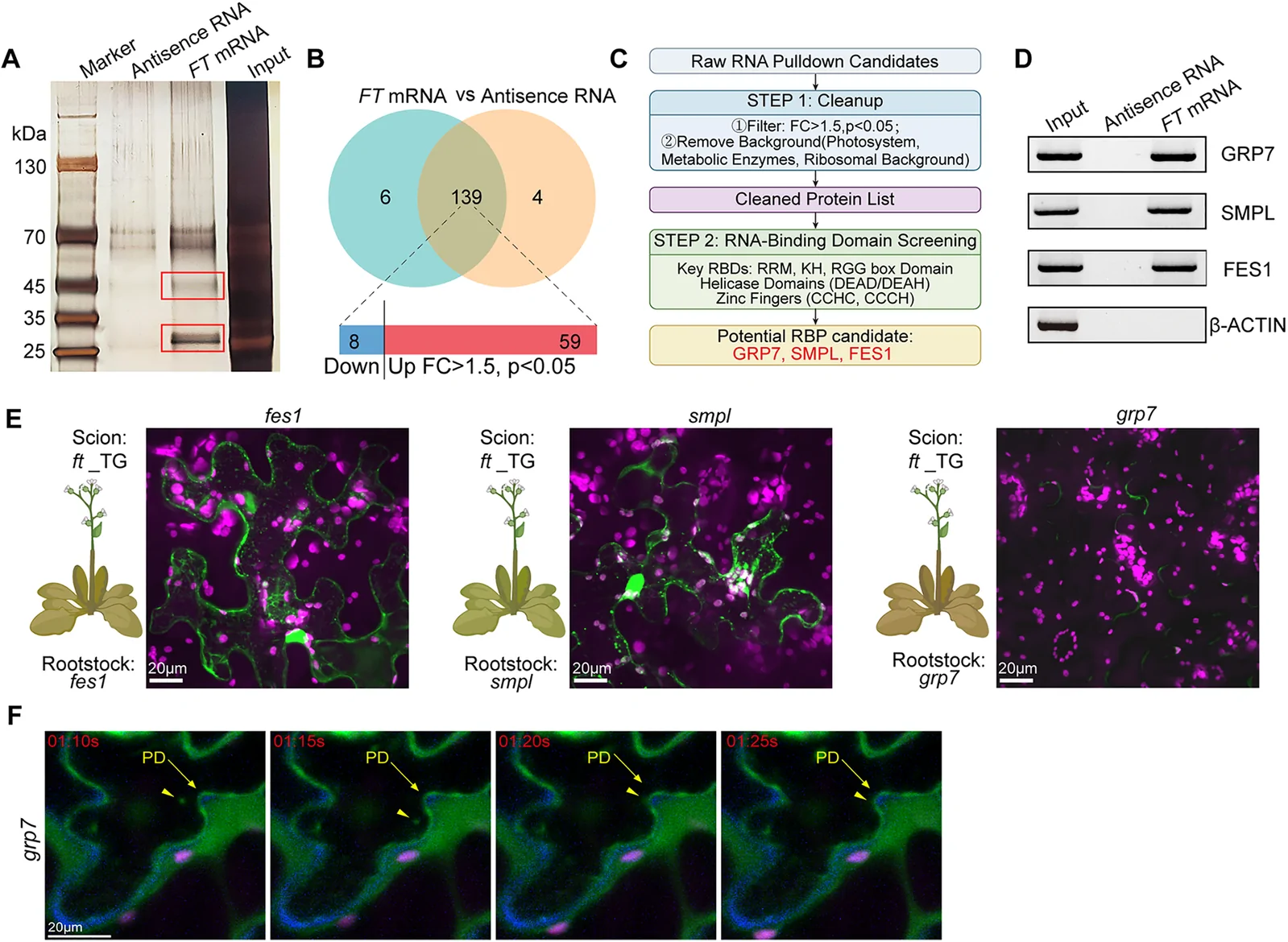
Functional assessment of candidate *FT* RNA-associated proteins in the grafting assay. **(A)** Identification of proteins enriched in the *FT* sense-RNA pull-down relative to the antisense-RNA control. The red box indicates differential bands. **(B)** Venn diagram and bar chart showing the differentially accumulated proteins in the *FT*-probe and antisense-probe groups identified by mass spectrometry. **(C)** Screening workflow for candidate *FT* RNA-associated proteins. **(D)** Immunoblot validation of candidate-protein enrichment in the *FT* sense-RNA pull-down. **(E)** Functional assessment of three candidate proteins in *FT* mRNA transport after grafting. Loss-of-function mutants for each candidate protein were used as rootstocks, and the *ft*_TG line was used as the scion. Scale bars, 20 μm. Yellow arrowheads indicate fluorescent RNA foci. **(F)** Time-lapse imaging of *FT* mRNA trafficking in the *grp7* background. The data underlying this figure can be found in S1 Data. The uncropped blot and gel images are provided in S1 Raw Images.

To assess their contributions to long-distance *FT* mRNA transport, we grafted the *ft*_TG line onto *grp7*, *fes1*, and *smpl* mutant rootstocks, respectively (Figs 7E and S7; S8 Table). *FT*-associated signals in *ft*_TG scions were strongly reduced when *grp7* rootstocks were used, supporting a contribution of GRP7 to efficient rootstock-to-scion *FT* mRNA transport.

To further illustrate the role of GRP7 in *FT* trafficking, we next examined *FT* trafficking dynamics in the *grp7* background. In *grp7*, *FT-*associated puncta still reached PDCB1-marked plasmodesmata-associated regions but remained at these regions, and no transfer event was observed during the 3-min imaging window (Fig 7F; S3 Video). These observations are consistent with a contribution of GRP7 to the intercellular transfer step following the association of *FT* puncta with plasmodesmata-associated regions.

Together, these results support a functional contribution of GRP7 to efficient *FT* mRNA transport, potentially during or after the association of *FT* puncta with plasmodesmata-associated regions.

## Discussion

Live-cell single-molecule imaging has become an important approach for resolving the spatiotemporal heterogeneity of gene expression [1,2]. Yet progress in plants has lagged behind animal systems because plant cells impose distinctive physical constraints on fluorescence-based detection. In particular, the large central vacuole compresses the cytoplasm into a thin peripheral layer, concentrates unbound probes, and generates substantial background signal [26,27]. Here, we addressed this challenge by combining CRISPR–dCas13X targeting with a plant-optimized conditional-stability module based on the cPIL degron. This design improves signal-to-noise while minimizing detectable perturbation to the endogenous transcript, thereby helping reconcile two central requirements for live RNA imaging in plants: sensitivity and physiological fidelity.

Several RNA-targeting CRISPR platforms could in principle be adapted for live-cell RNA imaging. Previous studies established CRISPR-based live-cell imaging using Cas9- and Cas13-derived architectures [17,22,28,29]. In plant cells, however, probe size is likely to be especially important because trafficking through plasmodesmata is constrained by strict size limits. Because our platform also incorporates a large amplification scaffold (24× SunTag), we selected the compact Cas13X (~445 aa) [30] as the RNA-recognition module. This was primarily an engineering choice, rather than a claim that dCas13X is universally superior for RNA imaging. By contrast, CRISPR-Csm has recently emerged as a powerful RNA imaging strategy, but its multi-protein architecture introduces additional complexity in expression and assembly [31,32]. We therefore view currently available systems as involving different trade-offs rather than a single best solution. Within this framework, dCas13X provided a rational starting point for a first-generation plant RNA imaging platform.

A central feature of this system is its binding-dependent stabilization mechanism. Instead of relying on exogenous induction systems such as AID [33] or split-fluorescent protein strategies [26], we engineered an autonomous switch in which the cPIL degron is positioned near the antigen-binding interface of the scFv. In this configuration, unbound probes are preferentially cleared, whereas target-associated probes are retained in a manner consistent with binding-dependent protection. In plant cells, where diffuse peripheral background is particularly problematic, this architecture actively reduces the free-probe pool while preserving fluorescence at bona fide RNA loci. The resulting improvement in signal-to-noise highlights the practical value of conditional probe destabilization for endogenous RNA imaging in plants.

Specificity and low perturbation are equally important for endogenous RNA imaging. Our system uses dCas13X and guide RNAs to recognize endogenous transcripts, and previous work has generally reported low off-target activity for dCas13-based RNA-targeting systems when guides are properly designed [29,30,34,35]. In our experiments, colocalization with the MS2/MCP reporter system further supported guide-dependent target recognition, whereas analyses of transcript abundance, protein level, and RNA decay did not reveal a detectable effect of the imaging system on the tested target RNA. The residual apparent colocalization observed in the non-targeting gRNA control may reflect background-level overlap rather than target-dependent recognition. In plant cells, the large central vacuole compresses the cytoplasm into a thin peripheral layer, increasing the effective local density of fluorescent puncta within a restricted cytoplasmic space and thereby increasing the probability of incidental overlap within the optical resolution limit. Segmentation thresholds and weak background fluorescence may further contribute to this residual apparent colocalization. Compared with smFISH, the principal advantage of the present platform is therefore not necessarily higher detection efficiency per se, but the ability to image endogenous RNAs in living plant cells with reduced background and to capture their spatial and temporal dynamics.

Applying this platform to *FT* mRNA provided biologically informative live-cell observations relevant to a long-standing question in plant biology. While FT protein is a well-established component of florigen [36,37], the extent to which *FT* mRNA itself can undergo systemic transport has remained debated. Previous grafting experiments provided evidence that *FT* mRNA moves systemically and contributes to floral regulation [38]. Separate live-cell studies showed that mobile mRNAs, including *FT*, are selectively targeted to plasmodesmata [27]. Our imaging extends this literature by directly visualizing *FT* mRNA association with plasmodesmata and by capturing cell-to-cell trafficking events in living plant cells. The kinetic analysis further suggests that *FT* mRNA movement is not simply diffusion-like, but instead includes at least two distinguishable phases: an initial residence phase at plasmodesmata-associated regions followed by rapid intercellular transit. In the grafting system, *FT* mRNA signals became detectable in the *ft* scion after grafting onto a wild-type stock, and the presence of full-length *FT* transcript in the scion was further supported by RT-PCR and sequencing. Together, these results support long-distance *FT* mRNA transport in our experimental system.

Beyond visualizing *FT* mRNA movement, our data implicate GRP7 in efficient *FT* mRNA transport. GRP7 was enriched in *FT* sense-RNA pull-downs, and *grp7* rootstocks showed substantially reduced detection of *FT* mRNA in grafted scions. Moreover, *FT* puncta in *grp7* cells still reached plasmodesmata-associated regions but did not cross the cell boundary during the 3-min observation window, a phenotype consistent with a role for GRP7 at or after plasmodesmata docking. AtGRP7 binds hundreds of cellular RNAs and regulates flowering through multiple pathways [39,40], while biochemical analyses indicate high affinity but limited sequence selectivity for long RNAs [41]. Consequently, our pull-down and genetic data do not establish an *FT*-specific interaction; GRP7 may bind *FT* RNA directly or may promote the formation, stability, or remodeling of a transport-competent *FT* ribonucleoprotein complex. Previous trafficking studies provide a broader context for this function. AtGRP7, designated AtSRBP1 by Yan and colleagues (2020), undergoes plasmodesmata-dependent cell-to-cell movement and binds single-stranded small RNAs [42], whereas CmPSRP1 denotes the pumpkin phloem protein that mediates systemic small-RNA delivery [43]. GRP7–Dronpa movement was detected across junctions between grafted Arabidopsis calli under light/dark conditions at the end of the day, when GRP7 expression is high [44]. Together with the circadian regulation of AtGRP7 expression, this finding suggests that the mobility or detectability of GRP7 may vary with tissue context and time of day. Whether GRP7 accompanies *FT* mRNA during vascular transport remains unknown.

Despite these advances, the present system still has limitations. The dCas13X–SunTag/GFP–cpScFv–cPIL complex remains relatively large, guide selection remains partly empirical because current design resources were developed primarily from Cas13d rather than Cas13X datasets, and the degron-stabilization strategy was validated here only in a GFP-based single-color configuration. Whether cPIL retains optimal behavior in other reporter contexts remains to be determined experimentally.

In summary, this work presents a versatile and high-fidelity platform for single-transcript-level RNA imaging in plants that addresses a central technical barrier imposed by plant cell architecture. By applying this tool to *FT* mRNA, we provide direct live-cell evidence consistent with plasmodesmata-associated trafficking, cell-to-cell transfer, and long-distance *FT* mRNA transport in our experimental system. Our data further support a functional contribution of GRP7 to efficient *FT* mRNA transport. More broadly, this study expands the toolkit available for investigating plant RNA dynamics and provides a framework for dissecting the mechanisms underlying long-distance RNA signaling in plants.

## Materials and methods

### Plant materials and growth conditions

*Nicotiana benthamiana* and *Arabidopsis thaliana* (Col-0) were grown in soil in a controlled greenhouse at 22 °C under long-day conditions (16 h light/8 h dark, light intensity ~120 µmol m^−2^ s^−1^). The *A. thaliana* mutant *ft-10 (CS9869)* was obtained from the Arabidopsis Biological Resource Center (ABRC). For plate-based assays, seeds were surface-sterilized and stratified at 4 °C for 2 d before sowing on half-strength Murashige and Skoog (1/2 MS) medium supplemented with 1% (w/v) sucrose and 0.8% (w/v) agar.

### Plasmid construction and *Agrobacterium* strain

All DNA fragments encoding the circularly permuted scFv mutants (mscFv) and candidate degron peptides were synthesized by Tsingke Biotechnology Co. (Beijing, China). All vectors were based on *pCAMBIA1300* derivatives. For the pUBQ10::mscFv constructs, the mscFv fragments were PCR-amplified and cloned downstream of the pUBQ10 promoter between the *Hind*III and *Nco*I restriction sites (S1 Table). For the *pUBQ10::mCherry-P2A-GFP-CPs* constructs, the degron peptide fragments were PCR-amplified and cloned into the C terminus of the mCherry–P2A–GFP cassette using homology-based recombination between the *Bam*HI and *Bsr*GI restriction sites. For the 10 degron peptides reported in previous studies, the corresponding oligonucleotides were synthesized (Tsingke), annealed at 95 °C for 5 min, and ligated into the same *Bam*HI *and Bsr*GI restriction sites at the GFP C terminus using T4 DNA ligase. To reduce fluorescence background, DNA sequences encoding GFP–cpScFv–cPIL and GFP–cpScFv were synthesized (Tsingke), PCR-amplified, and cloned downstream of the pUBQ10 promoter between the *Hind*III *and Nco*I restriction sites. For the MS2-based reporter, the 24× MS2 cassette was inserted into the 3′ UTR of *Actin2*, generating an *Actin2*–24× MS2 fusion construct driven by the *UBQ10* promoter. In the same plasmid, the DNA fragment encoding mCherry–MCP was also cloned under the control of the *UBQ10* promoter. For endogenous RNA imaging (including *NtACT7*, *ACT2*, *FT*, *IRX9*, *ATPP2-A1*, and *CORTEX4*), gRNA sequences were cloned into the *pUBQ10::dCas13X–SunTag–IRES–GFP–cpScFv–cPIL* construct, downstream of the *AtU6-26* promoter. To visualize plasmodesmata, the CS of the specific PD marker PDCB1 (AT5G61130, Plasmodesmata Callose-Binding Protein 1), fused to mTagBFP2, was cloned under the control of the *UBQ10* promoter. Unless otherwise stated, DNA polymerases, restriction endonucleases, ligases, and assembly mixes were purchased from New England Biolabs (NEB). All plasmids were verified by Sanger sequencing and introduced into *Agrobacterium tumefaciens* strain *GV3101* for subsequent transient expression or stable transformation.

### *Agrobacterium-*mediated infiltration of *Nicotiana benthamiana*

*Agrobacterium tumefaciens* strains were streaked onto LB agar plates supplemented with kanamycin (50 µg mL^−1^) and rifampicin (25 µg mL^−1^). Single colonies were inoculated into liquid LB medium containing the same antibiotics and grown overnight. Cells were harvested by centrifugation, resuspended in infiltration buffer (10 mM MES, pH 5.6; 10 mM MgCl_2_; 150 µM acetosyringone), and adjusted to an OD_600_ of approximately 0.6. After incubation for 2 h, the bacterial suspensions were infiltrated into the abaxial side of leaves of 3-week-old *N. benthamiana* plants using a needleless syringe. For co-infiltration, bacterial suspensions were mixed at a 1:1 (v/v) ratio. Plants were maintained in a growth chamber for 2 d after infiltration, and infiltrated leaf tissues were subsequently harvested for confocal microscopy.

### Proteome-wide degron screening (LC–MS/MS)

Leaves of five-week-old tobacco (*Nicotiana benthamiana*) were treated with 10 µM MG132 or a mock solution (1× PBS) for 24 h. After treatment, leaf tissues were subjected to liquid chromatography–tandem mass spectrometry (LC–MS/MS) analysis (Shanghai Personal Biotechnology Co., Shanghai, China). Total protein was extracted using the FASP method, digested with trypsin (1:50 w/w), and analyzed on an Orbitrap Astral mass spectrometer (Thermo Fisher Scientific) coupled to a Vanquish Neo LC system. Data were acquired in Data-Independent Acquisition (DIA) mode and processed using DIA-NN software (v.1.8). Differentially expressed proteins were identified based on a fold-change >2 and FDR <0.01.

### Assessment of degron-mediated degradation efficiency in *Nicotiana benthamiana*

To evaluate the degradation efficiency of candidate degron sequences, we cloned 10 previously reported protein degrons (S2 Table) as well as 90-nt fragments corresponding to 30 candidate C-terminal degron genes identified by proteomics into the C terminus of the GFP CDS in a 2A self-cleaving co-expression vector (S3 Table). This construct co-expresses a GFP-fusion reporter together with mCherry as an internal fluorescence control.

In addition, multiple truncated variants of CP13 were generated and inserted into the same reporter backbone (S4 Table). The resulting plasmids were introduced into *Nicotiana benthamiana* leaves by *Agrobacterium*-mediated infiltration. At 48 h post-infiltration, fluorescence signals were examined by fluorescence microscopy, and degron activity was quantified by comparing the GFP fluorescence intensity to the corresponding mCherry signal to normalize for expression variability.

### Degron-dependent turnover kinetics of GFP fusion proteins

GFP–cpScFv or GFP–cpScFv–cPIL was transiently expressed in *Nicotiana benthamiana* leaves by *Agrobacterium*-mediated infiltration under the control of the same promoter. At 36 h post-infiltration, when robust fluorescence was readily detectable in the positive control, chemical treatments were initiated. To assess protein turnover, de novo protein synthesis was inhibited with 100 µM cycloheximide (CHX) prepared in 1× PBS. Infiltrated leaf tissues were harvested from the same infiltrated areas at 0, 30, 60, and 90 min after CHX treatment, and the collected samples were used for subsequent immunoblot analysis.

### gRNA design and guide selection for dCas13X imaging

Guide RNAs were designed using a fixed Cas13X-compatible architecture consisting of the Cas13X direct repeat and a 30-nt spacer. For each target transcript, candidate target sites within the CDS were first identified in silico using Cas13design as a heuristic resource to prioritize regions with favorable predicted accessibility. For each target transcript, the five highest-scoring 23-nt candidate spacers predicted by Cas13design were selected for further processing. Each 23-nt candidate spacer was mapped back to the corresponding target transcript by reverse-complement alignment. The target site was then extended using authentic adjacent transcript sequence to generate a 30-nt Cas13X spacer. In this process, we prioritized designs in which the original 23-nt candidate core occupied positions 8–30 of the final 30-nt spacer, thereby retaining the Cas13design-predicted core within the region corresponding to the reported Cas13X crRNA 16–30 nt activity-sensitive region [30]. These candidates were then evaluated for predicted off-target risk by sequence-similarity analysis against the *Arabidopsis* TAIR10 transcript set. The candidate with the lowest predicted off-target risk was selected for gRNA construction and imaging validation. The total number of predicted target sites together with the guide scores, standardized guide scores, and predicted off-target information for the guides used in this work are provided in S5 Table.

### RT-PCR

Total RNA was isolated using a Plant RNA Extraction Kit (BIOFIT, RN33050) and treated to remove residual genomic DNA according to the manufacturer’s instructions. Equal amounts of RNA were reverse-transcribed into cDNA using a reverse transcription kit (abm, G592). Quantitative RT-PCR (qRT-PCR) was then performed with gene-specific primers synthesized for the target genes. Each sample was analyzed with at least three technical replicates, and expression levels were normalized to a stably expressed internal reference gene. Relative transcript abundance was calculated using the 2^−ΔΔCt^ method. Primer sequences are provided in S6 Table.

### Immunoblotting

Total protein was extracted from leaf tissues using extraction buffer (50 mM Tris-HCl, pH 7.5, 150 mM NaCl, 0.5% NP-40, 1 mM EDTA, and protease inhibitor cocktail). Lysates were clarified by centrifugation at 13,000*g*, separated by SDS–PAGE, and transferred to PVDF membranes. Membranes were probed with anti-GFP (1:5,000, Abbkine, ABT2020), anti-ACT2 (1:3,000, Agrisera), anti-GRP7 (1:2,000, PhytoAB, PHY3535A), anti-GAPDH (1:1,000, Agrisera), and anti-FLAG tag (1:1,000, Sigma) antibodies, followed by HRP-conjugated secondary antibodies. Signals were visualized using ECL substrate (Bio-Rad) and quantified using ImageJ. For stability assays, leaf discs were treated with 100 µM cycloheximide (CHX) or 50 µM MG132 and harvested at the indicated time points.

### Single-molecule fluorescence in situ hybridization (smFISH)

smFISH was performed using Cy3-labeled Stellaris probes (Biosearch Technologies) targeting the *ACT2* CDS, designed to exclude the dCas13X-binding region (S7 Table). A single construct co-expressing the dCas13X–SunTag/GFP–cpScFv–cPIL system and gRNA was transformed into *Arabidopsis*. Leaf tissues were fixed in 4% paraformaldehyde (PFA) in MES buffer (pH 5.7) for 30 min at room temperature. To facilitate permeabilization while maintaining tissue architecture, fixed samples were mounted on slides and processed via the freeze-cracking method using liquid nitrogen. After air-drying for 30 min, tissues were permeabilized in 70% ethanol with gentle agitation for ≥1 h and rehydrated in MES buffer. Hybridization was conducted overnight at 37 °C in a humidified dark chamber using 250 nM probes. Stringency washes were performed twice with 1× SSC containing 40% formamide at 37 °C for 45 min each to remove nonspecific binding. Samples were mounted in ProLong Diamond Antifade Mountant with DAPI (Invitrogen) for nuclear counterstaining prior to microscopy.

### Generation of transgenic lines and micrografting

Stable transgenic *Arabidopsis* lines were generated via the *Agrobacterium*-mediated floral dip method using strain *GV3101*. T1 transformants were selected on 1/2 MS plates containing 50 µg mL^−1^ kanamycin. Homozygous T3 lines with single-locus insertions were established for all experiments.

Micrografting was performed on 7-day-old seedlings grown vertically under short-day conditions (8 h light/16 h dark) to promote hypocotyl elongation. Scions were cut transversely and aligned with rootstocks using a silicone microtube (0.3 mm i.d.) for stabilization. Grafted plants were maintained in high humidity for 5–7 d to facilitate union formation. Adventitious roots were manually removed to ensure rootstock-dependent growth.

### RNA pull-down–LC–MS/MS assay

*FT* sense mRNA and an antisense transcript (negative control) were synthesized by in vitro transcription using the MEGAscript T7 kit (Invitrogen) following the manufacturer’s instructions. Purified RNAs were 3′-end-labeled with desthiobiotin (RNA 3′ Desthiobiotinylation Kit; Thermo Scientific) for streptavidin-mediated capture. The concentration of each labeled RNA was quantified, and its integrity was verified by denaturing agarose gel electrophoresis to ensure comparable bait quality between reactions.

Total protein lysates were prepared in pre-chilled Pierce IP Lysis Buffer (Thermo Scientific), cleared by centrifugation at 4 °C, and normalized to equal input amounts. Where indicated, lysates were pre-cleared with magnetic beads to reduce nonspecific binding. RNA pull-down was performed using the Pierce Magnetic RNA–Protein Pull-Down Kit (Thermo Scientific). Desthiobiotinylated RNA was immobilized on streptavidin magnetic beads and incubated with protein lysate at 4 °C for 6 h with gentle rotation. Beads were washed stringently according to the kit protocol, and bound proteins were eluted. Eluates were divided for LC–MS/MS identification and immunoblot validation. All procedures were conducted on ice or at 4 °C to preserve RNA–protein interactions.

### Microscope settings and image acquisition

Imaging was performed on an Olympus SpinSR10 spinning-disk confocal microscope equipped with a scanning unit and an EMCCD camera. A 63× oil-immersion objective (NA 1.2) was used for all quantification. GFP was excited at 488 nm (emission 500–550 nm), mCherry/Cy3 at 561 nm (emission 570–620 nm), and BFP at 405 nm (emission 440–450 nm). For Z-stacks, optical sections were acquired at 0.2 µm intervals. Laser power and exposure times were kept constant across all comparative experiments (typically 100–200 ms exposure).

### Image processing and quantification of single RNA puncta

Image analysis was performed using the Fiji software [45]. The signal-to-noise ratio was calculated as “SNR = I_(spot)/I_(cyto)”, where “I_(spot)” is the mean intensity of an RNA punctum and “I_(cyto)” is the mean intensity of the neighboring cytoplasm.

To quantify the number of RNA molecules per cell, 3D Z-stacks were first converted to two-dimensional images using maximum-intensity projection (MIP). Regions of interest (ROIs) corresponding to individual cells were manually segmented based on the brightfield images. For the analysis of root marker genes in Fig 5, cellular ROIs were selected from the anatomically corresponding cell type for each marker on the basis of root tissue morphology in the brightfield images, namely cortical cells for *CORTEX4*, xylem cells for *IRX9*, and phloem-domain cells for *ATPP2-A1*. Background subtraction was applied using a rolling-ball algorithm with a radius of 50 pixels to remove inhomogeneous background illumination. Single RNA spots were detected and counted using the TrackMate plugin [46] within Fiji. The Laplacian of Gaussian (LoG) detector was employed, which is optimized for diffraction-limited spot detection. The estimated blob diameter was set to 0.5 μm, corresponding to the theoretical resolution limit of the optical system, and an intensity threshold was applied to exclude background noise. The number of RNA spots within each cellular ROI was automatically calculated and exported for analysis.

### Quantitative reanalysis of the *FT* time-lapse imaging data

For dynamic analysis of *FT* mRNA trafficking, time-lapse image series were analyzed frame by frame in Fiji/ImageJ. A plasmodesmata-associated docking event was defined as the first frame in which an *FT* punctum became stably associated with the plasmodesmata-associated cell interface region. Dwell time was defined as the time interval between initial docking and the onset of intercellular transfer. A successful transfer event was defined as an *FT* punctum that moved from one cell side to the adjacent cell side across the same interface region and remained detectable after crossing. Transit time was defined as the interval between the onset of boundary crossing and completion of signal appearance on the adjacent cell side. Transfer frequency was calculated as the number of successful transfer events per minute per cell interface. In the *grp7* background, puncta that reached plasmodesmata-associated regions but did not cross the cell boundary during the 3-min imaging period were scored as non-transferring docking events.

### Statistical analysis

Statistical analyses were performed using GraphPad Prism 9.0 (GraphPad Software). Data are presented as mean ± s.d. unless otherwise stated. Comparisons between two groups were performed using a two-tailed Student *t* test. Multi-group comparisons were analyzed using one-way ANOVA with Tukey’s post hoc test. For each dataset, the sample size (“*n*”) and the nature of the quantified units are specified in the corresponding figure legend. Unless otherwise stated, “*n*” refers to biologically independent samples, such as independent plants, roots, grafts, infiltrations, or cells derived from independent biological samples. *P* < 0.05 was considered statistically significant. Significance levels are indicated as *P < 0.05, **P < 0.01, ***P < 0.001, and ****P < 0.0001.

## Supporting information

S1 Fig Representative fluorescence images of various C-terminal peptide tags. Images of *N. benthamiana* epidermal cells expressing GFP fused with various C- terminal peptide tags, related to Fig 2. Scale bars, 50 μm.
(TIF)

S2 Fig Screening of circularly permuted GFP–scFv mutants stabilized by dCas13X–SunTag. **(A)** Representative fluorescence images of *N. benthamiana* epidermal cells expressing circularly permuted scFv mutants (mscFv) alone, or co-expressing each mscFv mutant with dCas13X–SunTag. Scale bars, 50 μm. **(B)** Quantification of the average number of fluorescent puncta per cell in tobacco epidermal cells co-expressing dCas13X–SunTag with GFP–cpScFv or wild-type GFP–scFv. Each dot represents one cell, and bars indicate mean ± s.d. (*n* = 30 cells from 3 biologically independent infiltrations). The data underlying this figure can be found in S1 Data.
(TIF)

S3 Fig Colocalization analysis of dCas13X–SunTag (GFP) puncta with MCP–mCherry signals. Confocal fluorescent images showing overlay of dCas13X–SunTag (GFP) puncta with MCP–mCherry foci. Scale bars, 50 μm.
(TIF)

S4 Fig Effect of the GFP–cpScFv–cPIL system on *ACT2* RNA. **(A)** RT–qPCR analysis of *ACT2* mRNA abundance. Data are presented as mean ± s.d. (*n* = 3 biologically independent samples). **(B)** Immunoblot analysis of ACT2 protein levels (~42 kDa). GAPDH (~37 kDa) served as a loading control. **(C)** mRNA stability assay. *ACT2* mRNA decay was measured following actinomycin D treatment (*n* = 3 biologically independent samples, mean ± s.d.). The data underlying this figure can be found in S1 Data. The uncropped blot and gel images are provided in S1 Raw Images.
(TIF)

S5 Fig Time-lapse imaging capturing the cell-to-cell movement of *FT* mRNA in epidermal cells. Scale bar, 20 μm.
(TIF)

S6 Fig RT-PCR and sequencing validate long-distance transport of *FT* mRNA, related to Fig 6. (A) RT-PCR detection of *FT* mRNA. **(B)** Sanger sequencing confirmation of the *FT* amplicon obtained from *ft_*TG scions. The schematic indicates the position of the sequencing primer (7,037–7,060) and the covered region; representative chromatograms and sequence alignment show that the PCR product matches the *FT* transcript sequence, confirming that an intact and authentic *FT* mRNA is present in *ft_*TG scions. The uncropped blot and gel images are provided in S1 Raw Images.
(TIF)

S7 Fig Molecular identification of *Arabidopsis* T-DNA insertion mutants. **(A)** Genotyping of T-DNA insertion mutants by three-primer PCR. Primers were designed using the T-DNA Primer Design platform. For each sample, the left lane corresponds to the PCR product amplified with LP + RP, whereas the right lane corresponds to the product amplified with LB + RP. **(B)** RT-PCR analysis of the corresponding target gene transcripts in the mutants. The uncropped blot and gel images are provided in S1 Raw Images.
(TIF)

S1 Table Circularly permuted ScFv sequence. (XLSX)

S2 Table Amino acid sequences of C-terminal degrons previously reported in mammalian cells. (XLSX)

S3 Table The 30-aa C-terminal sequences of 30 proteins screened in *Nicotiana benthamiana.* (XLSX)

S4 Table Amino acid sequences of the CP13 truncation series. (XLSX)

S5 Table Design and screening of gRNA sequences suitable for Cas13X. (XLSX)

S6 Table RT-qPCR primers used to measure endogenous *AtACT2* transcript levels. (XLSX)

S7 Table smFISH probes used to target *AtACT2* mRNA. (XLSX)

S8 Table Primers used for mutant identification. (XLSX)

S1 Data Excel spreadsheet containing data values plotted in all main and supporting figures. (XLSX)

S1 Raw Images Original uncropped images supporting the gel, immunoblot, and silver-staining results in Figs 3, 7, S4, S6, and S7. (PDF)

S1 Video Time-lapse imaging showing cell-to-cell transfer of *FT* mRNA through PDCB1–BFP-labeled plasmodesmata. (AVI)

S2 Video Time-lapse imaging showing cell-to-cell transfer of *FT* mRNA. (MP4)

S3 Video Time-lapse imaging of *FT* mRNA trafficking in the *grp7* background. Plasmodesmata were labeled with PDCB1–BFP.
(MP4)

## Abbreviations

ABRC: Arabidopsis Biological Resource Center
CDR: complementarity-determining region
CDS: coding sequence
DAG: days after germination
DIA: Data-Independent Acquisition
cPIL: C-terminal Proteinase Inhibitor-Like degron
cpScFv: circularly permuted ScFv
CPs: C-terminal peptides
dCas13X: catalytically inactive Cas13X variants
LoG: Laplacian of Gaussian
MIP: maximum-intensity projection
NEB: New England Biolabs
PI: propidium iodide
ROIs: Regions of interest
smFISH: Single-molecule fluorescence in situ hybridization
SNR: signal-to-noise ratio

## References

[1] Hani S, Cuyas L, David P, Secco D, Whelan J, Thibaud M-C, et al. Live single-cell transcriptional dynamics via RNA labelling during the phosphate response in plants. Nat Plants. 2021;7(8):1050–64. doi: 10.1038/s41477-021-00981-3 34373603

[2] Guo A-Y, Zhang Y-M, Wang L, Bai D, Xu Y-P, Wu W-Q. Single-molecule imaging in living plant cells: A methodological review. Int J Mol Sci. 2021;22(10):5071. doi: 10.3390/ijms22105071 34064786 PMC8151321

[3] Alamos S, Reimer A, Niyogi KK, Garcia HG. Quantitative imaging of RNA polymerase II activity in plants reveals the single-cell basis of tissue-wide transcriptional dynamics. Nat Plants. 2021;7(8):1037–49. doi: 10.1038/s41477-021-00976-0 34373604 PMC8616715

[4] Gerber A, van Otterdijk S, Bruggeman FJ, Tutucci E. Understanding spatiotemporal coupling of gene expression using single molecule RNA imaging technologies. Transcription. 2023;14(3–5):105–26. doi: 10.1080/21541264.2023.2199669 37050882 PMC10807504

[5] Hu Y, Xu J, Gao E, Fan X, Wei J, Ye B, et al. Enhanced single RNA imaging reveals dynamic gene expression in live animals. Elife. 2023;12:e82178. doi: 10.7554/eLife.82178 36867026 PMC10032653

[6] Fukaya T, Lim B, Levine M. Enhancer control of transcriptional bursting. Cell. 2016;166(2):358–68. doi: 10.1016/j.cell.2016.05.025 27293191 PMC4970759

[7] Buxbaum AR, Haimovich G, Singer RH. In the right place at the right time: Visualizing and understanding mRNA localization. Nat Rev Mol Cell Biol. 2015;16(2):95–109. doi: 10.1038/nrm3918 25549890 PMC4484810

[8] Dynes JL, Steward O. Arc mRNA docks precisely at the base of individual dendritic spines indicating the existence of a specialized microdomain for synapse-specific mRNA translation. J Comp Neurol. 2012;520(14):3105–19. doi: 10.1002/cne.23073 22350812 PMC5546873

[9] Kuffner CJ, Marzilli AM, Ngo JT. RNA-stabilized coat proteins for sensitive and simultaneous imaging of distinct single mRNAs in live cells. Nat Methods. 2026;23(1):153–64. doi: 10.1038/s41592-025-02782-4 40983766 PMC12632079

[10] Bertrand E, Chartrand P, Schaefer M, Shenoy SM, Singer RH, Long RM. Localization of ASH1 mRNA particles in living yeast. Mol Cell. 1998;2(4):437–45. doi: 10.1016/s1097-2765(00)80143-4 9809065

[11] Lu S, Hou Y, Zhang X-E, Gao Y. Live cell imaging of DNA and RNA with fluorescent signal amplification and background reduction techniques. Front Cell Dev Biol. 2023;11:1216232. doi: 10.3389/fcell.2023.1216232 37342234 PMC10277805

[12] Kordyś M, Sen R, Warkocki Z. Applications of the versatile CRISPR-Cas13 RNA targeting system. Wiley Interdiscip Rev RNA. 2022;13(3):e1694. doi: 10.1002/wrna.1694 34553495

[13] Perčulija V, Lin J, Zhang B, Ouyang S. Functional features and current applications of the RNA-targeting type VI CRISPR-Cas systems. Adv Sci (Weinh). 2021;8(13):2004685. doi: 10.1002/advs.202004685 34254038 PMC8209922

[14] Kavuri NR, Ramasamy M, Qi Y, Mandadi K. Applications of CRISPR/Cas13-based RNA editing in plants. Cells. 2022;11(17):2665. doi: 10.3390/cells11172665 36078073 PMC9454418

[15] Tanenbaum ME, Gilbert LA, Qi LS, Weissman JS, Vale RD. A protein-tagging system for signal amplification in gene expression and fluorescence imaging. Cell. 2014;159(3):635–46. doi: 10.1016/j.cell.2014.09.039 25307933 PMC4252608

[16] Khuperkar D, Hoek TA, Sonneveld S, Verhagen BMP, Boersma S, Tanenbaum ME. Quantification of mRNA translation in live cells using single-molecule imaging. Nat Protoc. 2020;15(4):1371–98. doi: 10.1038/s41596-019-0284-x 32076351

[17] Chen M, Sui T, Yang L, Qian Y, Liu Z, Liu Y, et al. Live imaging of RNA and RNA splicing in mammalian cells via the dcas13a-SunTag-BiFC system. Biosens Bioelectron. 2022;204:114074. doi: 10.1016/j.bios.2022.114074 35149451

[18] Ogawa Y, Nishimura K, Obara K, Kamura T. Development of AlissAID system targeting GFP or mCherry fusion protein. PLoS Genet. 2023;19(6):e1010731. doi: 10.1371/journal.pgen.1010731 37315088 PMC10266622

[19] Nabet B, Roberts JM, Buckley DL, Paulk J, Dastjerdi S, Yang A, et al. The dTAG system for immediate and target-specific protein degradation. Nat Chem Biol. 2018;14(5):431–41. doi: 10.1038/s41589-018-0021-8 29581585 PMC6295913

[20] Nishimura K, Fukagawa T, Takisawa H, Kakimoto T, Kanemaki M. An auxin-based degron system for the rapid depletion of proteins in nonplant cells. Nat Methods. 2009;6(12):917–22. doi: 10.1038/nmeth.1401 19915560

[21] Cao H, Wang Y, Zhang N, Xia S, Tian P, Lu L, et al. Progress of CRISPR-Cas13 mediated live-cell RNA imaging and detection of RNA-protein interactions. Front Cell Dev Biol. 2022;10:866820. doi: 10.3389/fcell.2022.866820 35356276 PMC8959342

[22] Tang H, Peng J, Peng S, Wang Q, Jiang X, Xue X, et al. Live-cell RNA imaging using the CRISPR-dCas13 system with modified sgRNAs appended with fluorescent RNA aptamers. Chem Sci. 2022;13(47):14032–40. doi: 10.1039/d2sc04656c 36540819 PMC9728512

[23] Denyer T, Ma X, Klesen S, Scacchi E, Nieselt K, Timmermans MCP. Spatiotemporal developmental trajectories in the *Arabidopsis* root revealed using high-throughput single-cell RNA sequencing. Dev Cell. 2019;48(6):840-852.e5. doi: 10.1016/j.devcel.2019.02.022 30913408

[24] Shahan R, Hsu C-W, Nolan TM, Cole BJ, Taylor IW, Greenstreet L, et al. A single-cell *Arabidopsis* root atlas reveals developmental trajectories in wild-type and cell identity mutants. Dev Cell. 2022;57(4):543-560.e9. doi: 10.1016/j.devcel.2022.01.008 35134336 PMC9014886

[25] Shulse CN, Cole BJ, Ciobanu D, Lin J, Yoshinaga Y, Gouran M, et al. High-throughput single-cell transcriptome profiling of plant cell types. Cell Rep. 2019;27(7):2241-2247.e4. doi: 10.1016/j.celrep.2019.04.054 31091459 PMC6758921

[26] Huang N-C, Luo K-R, Yu T-S. Development of a split fluorescent protein-based RNA live-cell imaging system to visualize mRNA distribution in plants. Plant Methods. 2022;18(1):15. doi: 10.1186/s13007-022-00849-3 35130941 PMC8822845

[27] Luo K-R, Huang N-C, Yu T-S. Selective targeting of mobile mRNAs to plasmodesmata for cell-to-cell movement. Plant Physiol. 2018;177(2):604–14. doi: 10.1104/pp.18.00107 29581179 PMC6001314

[28] Wang H, Nakamura M, Abbott TR, Zhao D, Luo K, Yu C, et al. CRISPR-mediated live imaging of genome editing and transcription. Science. 2019;365(6459):1301–5. doi: 10.1126/science.aax7852 31488703 PMC13030913

[29] Yang L-Z, Wang Y, Li S-Q, Yao R-W, Luan P-F, Wu H, et al. Dynamic imaging of RNA in living cells by CRISPR-Cas13 systems. Mol Cell. 2019;76(6):981-997.e7. doi: 10.1016/j.molcel.2019.10.024 31757757

[30] Xu C, Zhou Y, Xiao Q, He B, Geng G, Wang Z, et al. Programmable RNA editing with compact CRISPR-Cas13 systems from uncultivated microbes. Nat Methods. 2021;18(5):499–506. doi: 10.1038/s41592-021-01124-4 33941935

[31] Colognori D, Trinidad M, Doudna JA. Precise transcript targeting by CRISPR-Csm complexes. Nat Biotechnol. 2023;41(9):1256–64. doi: 10.1038/s41587-022-01649-9 36690762 PMC10497410

[32] Xia C, Colognori D, Jiang XS, Xu K, Doudna JA. Single-molecule live-cell RNA imaging with CRISPR-Csm. Nat Biotechnol. 2025;43(12):2023–30. doi: 10.1038/s41587-024-02540-5 39966655 PMC12700784

[33] Saito Y, Kanemaki MT. Targeted Protein depletion using the auxin-inducible degron 2 (AID2) system. Curr Protoc. 2021;1(8):e219. doi: 10.1002/cpz1.219 34370399

[34] Wen H-P, Yu C, Bi S, Jiang L-H, Wang Z-G, Yao Z, et al. Programmable targeted RNA degradation via dCas13d-directed chaperone-mediated autophagy (dCasCMA). Nat Commun. 2025;16(1):10738. doi: 10.1038/s41467-025-65768-y 41315225 PMC12663475

[35] Yu L, Alariqi M, Li B, Hussain A, Zhou H, Wang Q, et al. CRISPR/dCas13(Rx) derived RNA N6-methyladenosine (m6A) dynamic modification in plant. Adv Sci (Weinh). 2024;11(39):e2401118. doi: 10.1002/advs.202401118 39229923 PMC11497087

[36] Corbesier L, Vincent C, Jang S, Fornara F, Fan Q, Searle I, et al. FT protein movement contributes to long-distance signaling in floral induction of *Arabidopsis*. Science. 2007;316(5827):1030–3. doi: 10.1126/science.1141752 17446353

[37] Tamaki S, Matsuo S, Wong HL, Yokoi S, Shimamoto K. Hd3a protein is a mobile flowering signal in rice. Science. 2007;316(5827):1033–6. doi: 10.1126/science.1141753 17446351

[38] Lu K-J, Huang N-C, Liu Y-S, Lu C-A, Yu T-S. Long-distance movement of *Arabidopsis* FLOWERING LOCUS T RNA participates in systemic floral regulation. RNA Biol. 2012;9(5):653–62. doi: 10.4161/rna.19965 22614833

[39] Meyer K, Köster T, Nolte C, Weinholdt C, Lewinski M, Grosse I, et al. Adaptation of iCLIP to plants determines the binding landscape of the clock-regulated RNA-binding protein AtGRP7. Genome Biol. 2017;18(1):204. doi: 10.1186/s13059-017-1332-x 29084609 PMC5663106

[40] Steffen A, Elgner M, Staiger D. Regulation of flowering time by the RNA-binding proteins AtGRP7 and AtGRP8. Plant Cell Physiol. 2019;60(9):2040–50. doi: 10.1093/pcp/pcz124 31241165

[41] Lühmann KL, Seemann S, Martinek N, Ostendorp S, Kehr J. The glycine-rich domain of GRP7 plays a crucial role in binding long RNAs and facilitating phase separation. Sci Rep. 2024;14(1):16018. doi: 10.1038/s41598-024-66955-5 38992080 PMC11239674

[42] Yan Y, Ham B-K, Chong YH, Yeh S-D, Lucas WJ. A plant SMALL RNA-BINDING PROTEIN 1 family mediates cell-to-cell trafficking of RNAi signals. Mol Plant. 2020;13(2):321–35. doi: 10.1016/j.molp.2019.12.001 31812689

[43] Ham BK, Li G, Jia W, Leary JA, Lucas WJ. Systemic delivery of siRNA in pumpkin by a plant PHLOEM SMALL RNA-BINDING PROTEIN 1-ribonucleoprotein complex. Plant J. 2014;80(4):683–94. 10.1111/tpj.12662 25227635

[44] Hasbioğlu Y, Weber S, Kragler F, Machin FQ. *Arabidopsis* callus grafts: A system to study symplasmic intercellular macromolecular transport and gene silencing spread. Plant J. 2023;115(2):301–16. doi: 10.1111/tpj.16326 37243907

[45] Schindelin J, Arganda-Carreras I, Frise E, Kaynig V, Longair M, Pietzsch T, et al. Fiji: An open-source platform for biological-image analysis. Nat Methods. 2012;9(7):676–82. doi: 10.1038/nmeth.2019 22743772 PMC3855844

[46] Tinevez J-Y, Perry N, Schindelin J, Hoopes GM, Reynolds GD, Laplantine E, et al. TrackMate: An open and extensible platform for single-particle tracking. Methods. 2017;115:80–90. doi: 10.1016/j.ymeth.2016.09.016 27713081

