# Supplementary material for: A binding-activated CRISPR/dCas13X imaging system enables precise RNA tracking in plants
Source: PLoS Biol. 2026 Oct 6;24(10):e3004043. doi: 10.1371/journal.pbio.3004043 (PMC13667925; doi:10.1371/journal.pbio.3004043)

Fig 3H

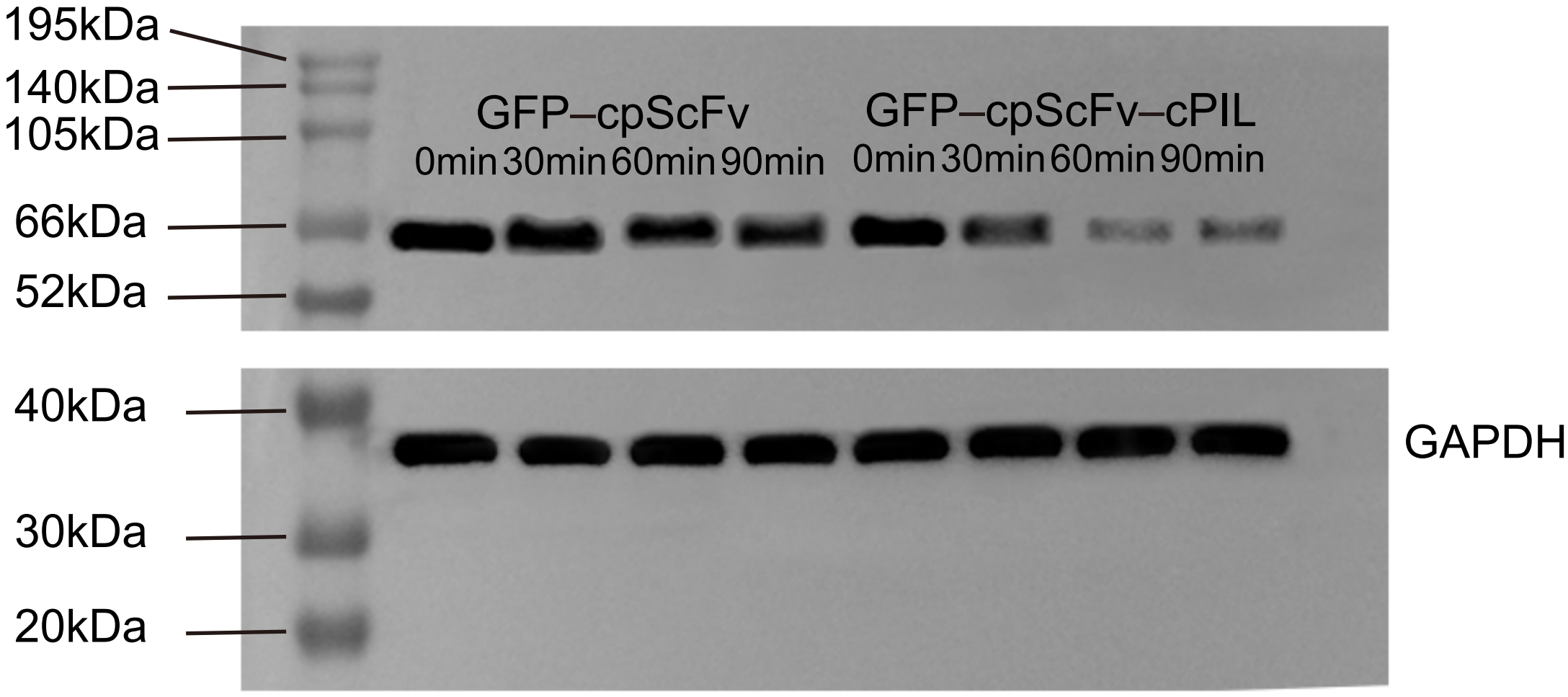

Fig 3I

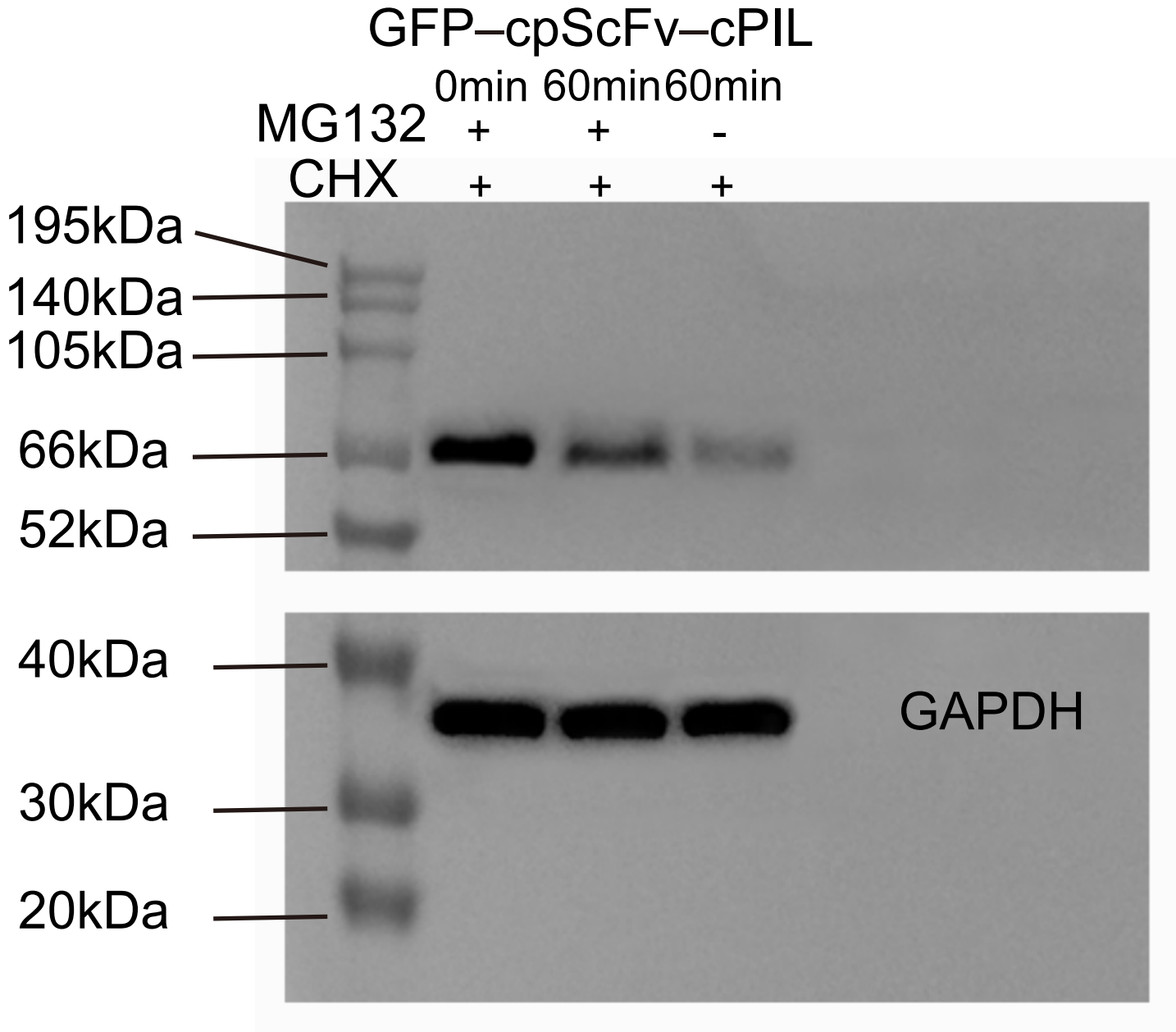

Fig 7A

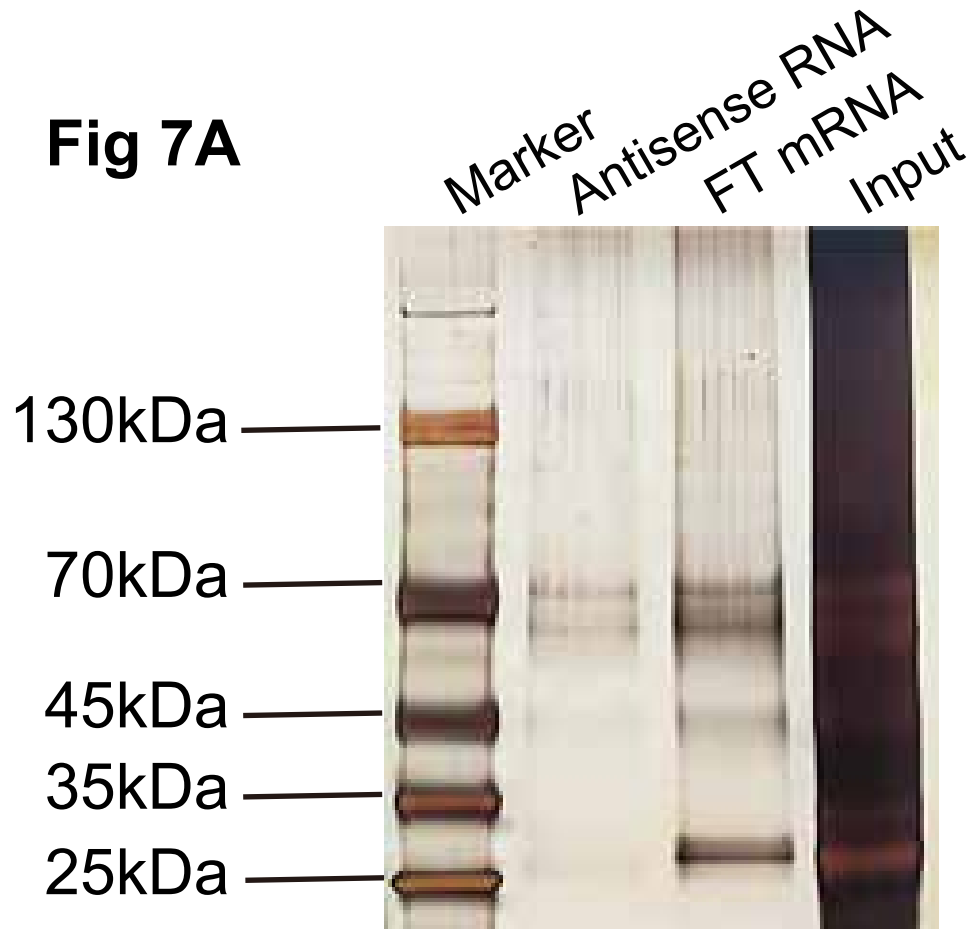

Fig 7D

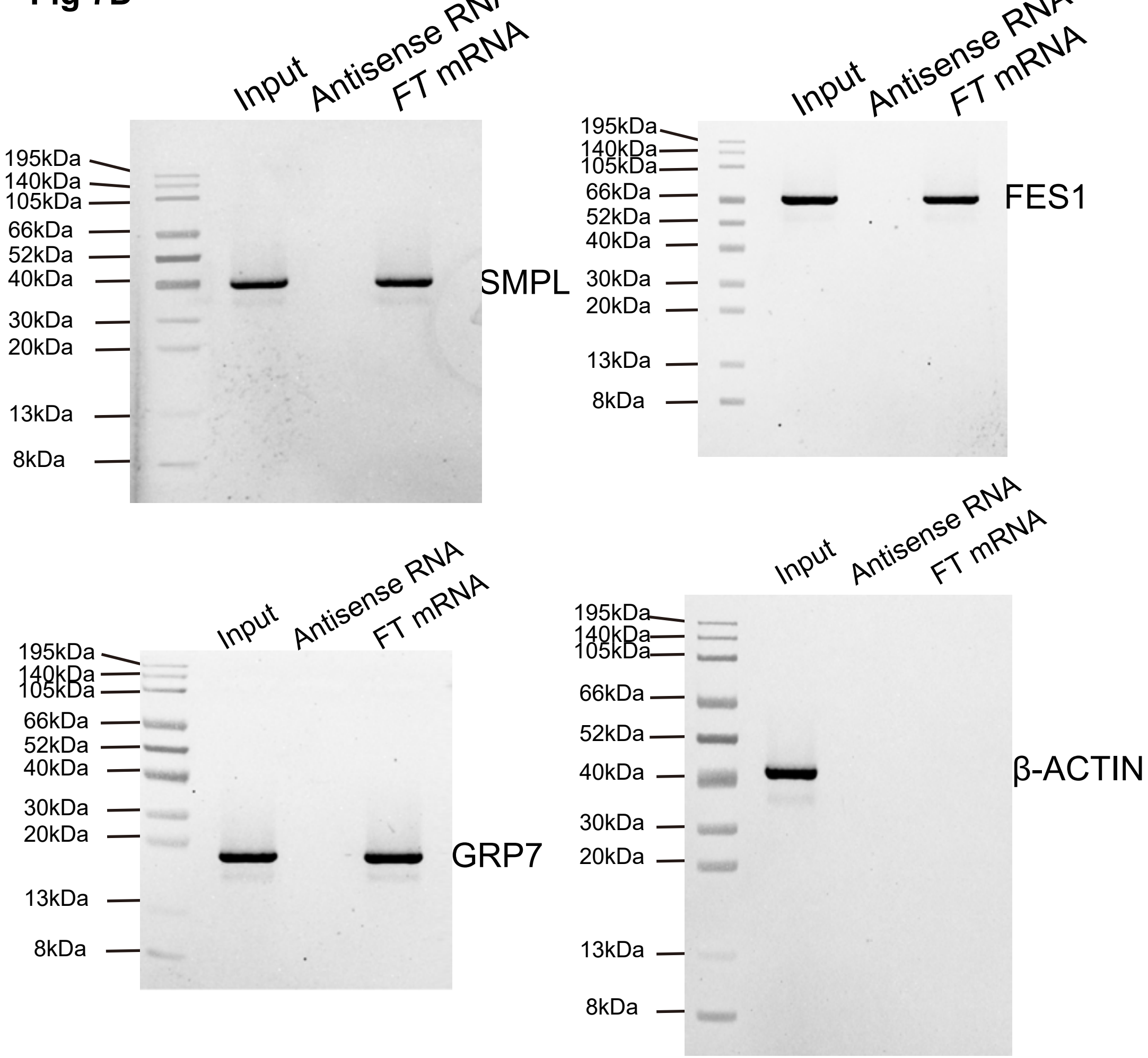

S4B Fig

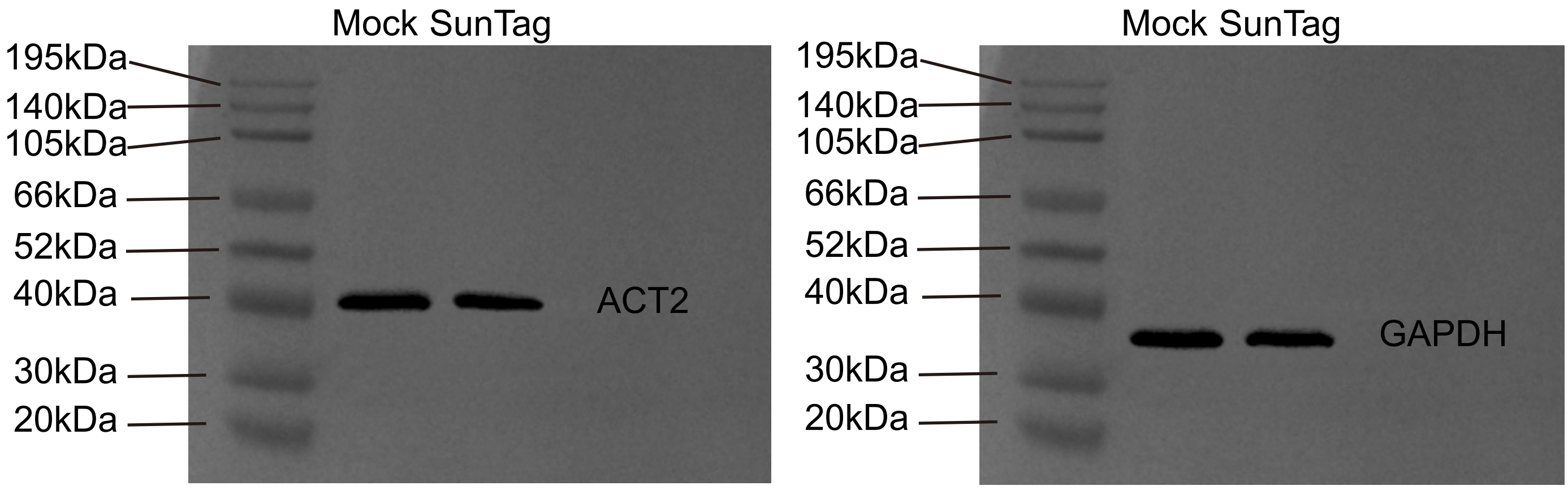

S6A Fig

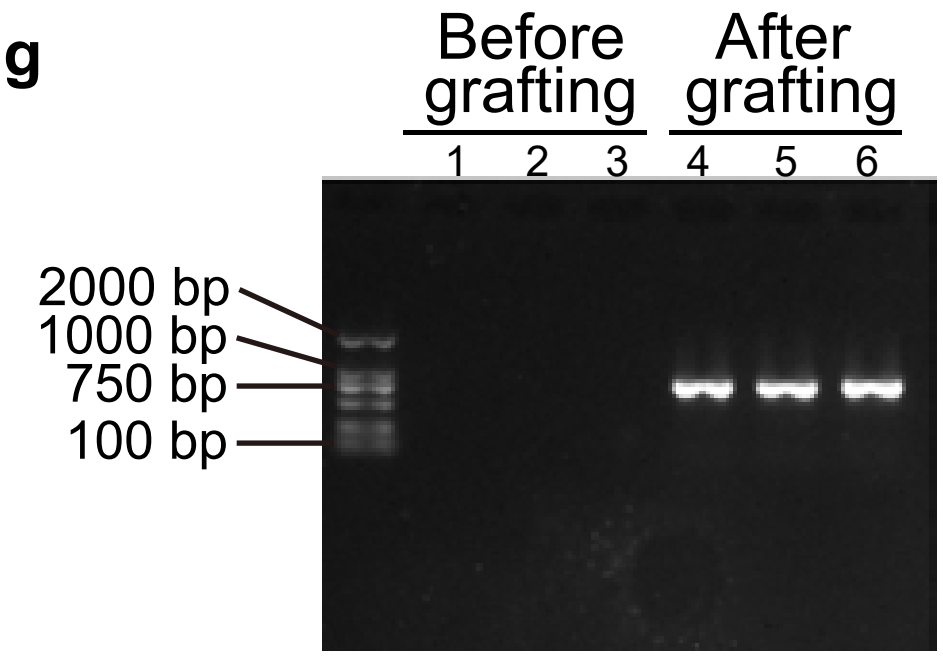

S7A Fig

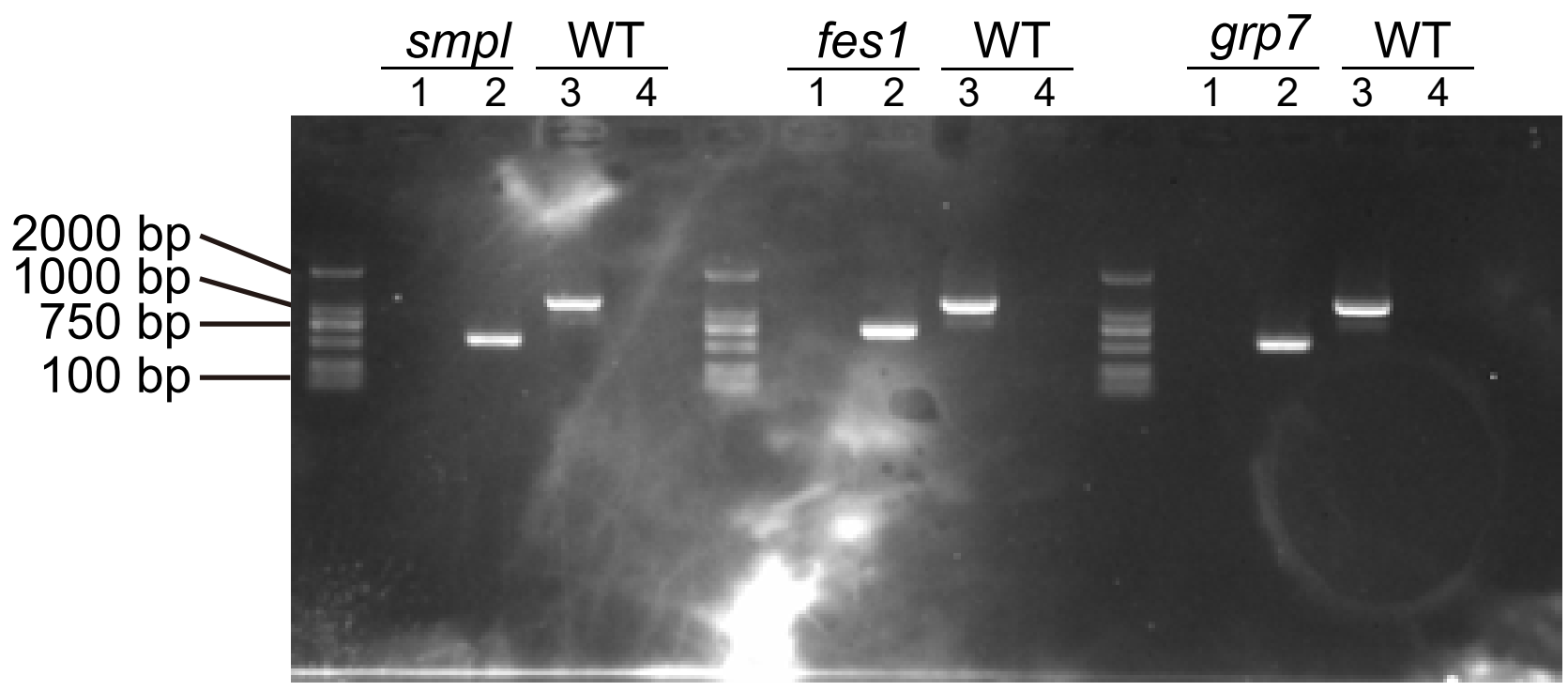

S7B Fig

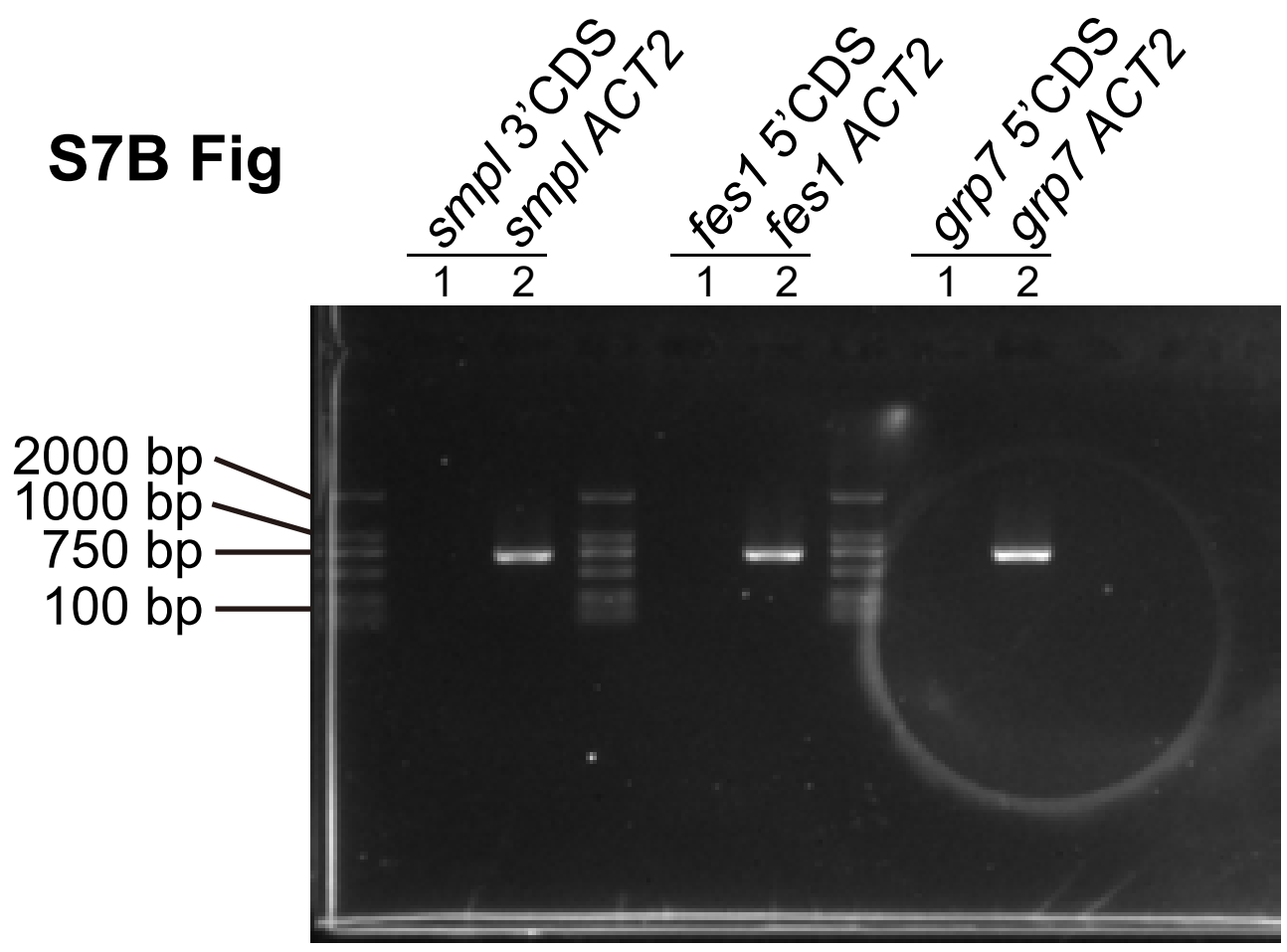

Supplement: Supplementary file 1 [file pbio.3004043.s017.pdf]
